# Impact of the Threat of COVID-19 Infections on the Perceived Risk to HPV Vaccination

**DOI:** 10.3390/vaccines10050829

**Published:** 2022-05-23

**Authors:** Yumi Shimizu, Kei Hirai, Yutaka Ueda, Asami Yagi, Fumio Ohtake

**Affiliations:** 1 Department of Clinical Psychology, Graduate School of Human Sciences, Osaka University, 1-2, Yamadaoka, Suita 565-0871, Osaka, Japan; u470822g@ecs.osaka-u.ac.jp; 2 Department of Obstetrics and Gynecology, Graduate School of Medicine, Osaka University, 2-2, Yamadaoka, Suita 565-0871, Osaka, Japan; y.ueda@gyne.med.osaka-u.ac.jp (Y.U.); a.yagi@gyne.med.osaka-u.ac.jp (A.Y.); 3 Department of Economics, Graduate School of Economics, Osaka University, 1-7, Machikaneyama-cho, Toyonaka 560-0043, Osaka, Japan; ohtake@econ.osaka-u.ac.jp

**Keywords:** cervical cancer, HPV vaccine, COVID-19 vaccine, vaccination status, vaccine willingness, health consciousness, health belief model

## Abstract

Vaccination rates for human papillomavirus (HPV) in Japan are significantly lower than other countries, and Japanese people are reluctant to be vaccinated. Repeated daily reports of COVID-19 infections and restrictions have made people more health conscious and aware of the danger of infectious diseases. In this study, we used the health belief model (HBM) to examine perceived threats of cancer and infectious diseases and to ascertain whether the new COVID-19 vaccination in addition to these perceived threats would increase vaccination intention against cervical cancer. We conducted a cluster analysis to classify the segmentation regarding the perceived threat, and a logistic regression analysis to predict factors influencing people accepting vaccination. We received 1257 completed surveys during our research. We classified the participants into six clusters, and the logistic regression analysis indicated eight factors significantly associated with the willingness to get the HPV vaccine: reliable information sources such as doctors and social networking sites (SNS), the recognition of COVID-19 symptoms, the awareness of COVID-19 vaccination, the importance of HPV prevention through vaccination, one’s own intention of COVID-19 vaccination, their intention of COVID-19 vaccination toward children, and benefits of HPV vaccination. Further research on HPV and COVID-19 vaccination is encouraged.

## 1. Introduction

While COVID-19 has attracted global attention, there are still some public health issues which remain unresolved in Japan. Cervical cancer is one such issue. Approximately 570,000 cases of, and 310,000 deaths owing to, cervical cancer are reported annually worldwide [1]. In Japan, there are about 10,000 patients with cervical cancer every year; cases have been detected earliest in the late 20s age group and seem to peak in the 50s age group [2]. Accordingly, in 2013, the Ministry of Health, Labour and Welfare (MHLW) decided to declare and administer the human papillomavirus (HPV) vaccine as a routine vaccination. However, due to excessive media reporting on some cases of alleged adverse reactions, after just three months of the vaccine’s formal inclusion in Japan’s national immunization programs, the “suspension of active recommendation” was enforced. Due to these circumstances, HPV vaccination rates have declined from 68.4–74.0% in the 1994–98 birth cohort to 0.6% in the 2000 birth cohort [3]; in 2018, the HPV vaccination rate was only 1.3% [4]. On the other hand, in 2020, individual guidance excluding active recommendation began to be provided and the vaccination rate slightly increased [5]. In November 2021, it was decided to withdraw the suspension of active recommendations, and these are scheduled to resume in April 2022. However, a tendency to take a negative view toward HPV vaccination persists. It is assumed that past cessation of proactive recommendation and social media advocacy led to Japanese people developing a strongly negative attitude toward not only HPV vaccination but also general vaccinations [3,6].

The health belief model (HBM) [7,8] and the theory of planned behavior can be applied to consider the intention to be vaccinated against cervical cancer. The HBM is a theoretical model, which attempts to explain and predict health behavior. It can be used to guide health promotion and disease-prevention programs wherein health behavior facilitators comprise perceived threat, perceived seriousness, perceived susceptibility, and perceived benefits and barriers to preventive behavior. Based on the HBM, the intention to get vaccinated will increase under the following conditions: the susceptibility and seriousness of cervical cancer incidence are high; the preventive benefits of HPV vaccination are high; the barriers to HPV vaccination are low; the subjective norm that “numerous people have been vaccinated” is high. Further, if the intention to be vaccinated increases, vaccination rates will also increase. However, repeated reports of adverse reactions—of the cervical cancer vaccine—in social media have heightened anxiety, fear, and avoidance of the vaccine. As a result, the barrier to the vaccine has become larger, and resistance to receiving the vaccine has increased. In the absence of recommendations to receive the vaccine, the normative awareness of vaccination has decreased [9]. Additionally, psychological reactance against the government, which had been promoting vaccination can also be considered a major barrier [10]. In other words, it is necessary to explain vaccination intentions structurally, by considering the perceived risk of cervical cancer in relation to existing social norms as well as threats to vaccination campaigns.

Meanwhile, COVID-19 infections have proliferated worldwide; in Japan, a state of emergency was first declared in April 2020. The perceived threat of COVID-19 has increased, owing to behavioral restrictions and repeated media reporting; the subjective norm “people must be vaccinated” has also intensified due to the recognition of the advantages as well as disadvantages of vaccination. The widespread use of vaccination in 2021 has thus allowed for some control. In fact, the vaccination has been progressing in Japan, and the vaccination of younger generations has been increasing because of an increased awareness of susceptibility caused by the epidemic of a variant. Furthermore, the increased willingness to be vaccinated against COVID-19 has significantly impacted vaccination readiness for other vaccines, including the HPV vaccine. In other words, people with a high intention to be vaccinated against COVID-19 may also have a high intention to be vaccinated against cervical cancer. However, in contrast, people who are averse to the COVID-19 vaccine are expected to have an even stronger aversion to the HPV vaccine.

Until now, studies have neglected whether the vaccination intention against COVID-19 has an impact on the vaccination intention against cervical cancer. Previous studies often focused on the negative impacts on the relation of COVID-19 with HPV vaccines and reported that COVID-19 has disrupted the delivery of HPV vaccination and has actually decreased HPV vaccination rate [11,12]. In contrast, one study researching the acceptability of COVID-19 among women found that HPV and annual flu vaccine recipients were positively associated with the acceptance of COVID-19 vaccine at a doctor’s recommendation [13]. In light of the previous study, Japanese people are expected to be pessimistic about COVID-19 vaccination because only few Japanese people have had HPV vaccine experience. However, recent information manipulation and exposure to new threats from COVID-19 will increase the willingness to be vaccinated against COVID-19, and the willingness of HPV vaccination will increase in proportion to this. Thus, our study focused on Japan where the number of HPV vaccine recipients is low and examined the intention to receive the HPV vaccine excluding the effect of previous vaccination experience.

In this study, we will first analyze and classify the patterns of threat perception, comprising perceived susceptibility and seriousness toward cervical cancer, other cancers, and COVID-19, among parents whose children are eligible for HPV vaccination. Second, using the classified perceived threat as a variable for the HBM, this study further examines whether a multivariate model—including variables such as social norms, disease literacy, psychological reactance, and the vaccination intention against the new COVID-19 vaccine—can explain the intention to vaccinate against cervical cancer.

## 2. Materials and Methods

### 2.1. Participants and Procedure

In January 2021, an online survey was administered to individuals—in the 20–80 age range with daughters aged 12–16 years—registered with a research company. We received 1257 responses. The respondents included 423 males aged 31–69 (*M* = 48.45, *SD* = 6.07) and 834 females aged 28–58 (*M* = 43.88, *SD* = 5.05). The survey was administered by a research company, and anonymized data were used for analysis.

### 2.2. Questionnaire

The main questionnaire items are shown in Table 1, and all detailed items are shown in Table S1. Each item was developed with reference to the data obtained from the interviews [14], and was extracted after discussion with a licensed psychologist, a clinical psychologist, and an obstetrician/gynecologist. In line with the HBM, items on COVID-19 and cervical cancer were also included in the questionnaire.

### 2.3. Statistical Analysis

Since cancer types differed in men and women (men: stomach, lung, and colorectal cancer; women: breast, cervical, and colorectal cancer), the values for each type were standardized and integrated into a single value for “cancer.” The COVID-19 data which were also separated by gender were standardized and merged into one value “COVID-19.” Next, descriptive statistics regarded each perceived threat toward cancer and COVID-19. Then, correlation analysis was conducted to reveal the relationship between each perceived threat toward cancer and COVID-19.

Second, this study conducted cluster analysis on the perceived seriousness and perceived susceptibility toward cervical cancer and COVID-19. This study integrated the cancer and COVID-19 data, performed cluster analysis, and examined the characteristics of each cluster.

Finally, a multivariable logistic regression analysis (forced enter and forward selection with likelihood ratio) was applied to examine whether the intention to HPV vaccination can be predicted from other variables, such as the HBM included in each cluster. Each of the 5 items concerning benefits of and resistance toward HPV and COVID-19 vaccination and cancer screening are also standardized and integrated into six values each. To conduct logistic regression analysis, dummy items for both the nominal scale and vaccination intention were created.

All analyses were conducted using IBM SPSS Statistics (ver. 26). *p* < 0.05 was considered statistically significant.

### 2.4. Informed Consent and Ethical Approval

The survey was conducted after obtaining approval from the Ethics Committee of the Department of Education, Graduate School of Human Sciences, Osaka University (No. 20067). All participants signed up with the research company prior to the beginning of the online survey, at which point they have already given their informed consent. The participants who fulfilled the conditions required by the research company read an explanation of the purpose of the survey and were informed that they could withdraw from the survey at any time. Their response constitutes their consent to participate in the survey. This process has been approved by the Ethics Committee.

## 3. Results

### 3.1. Participant Characteristics

The attributes of the participants are shown in Table 2. Of the total respondents, 66% underwent cancer screening, 81.8% had been vaccinated against influenza, and 3.1% of female subjects had received the HPV vaccine. Further, 65.7% and 67.7% of respondents would consider—for their children—the COVID-19 and HPV vaccine, respectively.

### 3.2. Correlation of Susceptibility and Seriousness toward Cancer and COVID-19

The correlation analysis was conducted for self-susceptibility/child-susceptibility and self-seriousness/child-seriousness toward cancer and COVID-19 (Table 3). Correlation analysis is a technique that reveals the relationship between variables, that is, how similar the movements of two variables are. It means that if susceptibility or seriousness in cancer is high, the susceptibility and seriousness of COVID-19 is also high. Pearson’s correlation coefficient was calculated, and significant correlations were found in all values. This clearly shows that there is an association between each of the variables.

### 3.3. Cluster Analysis

**Figure 1:**
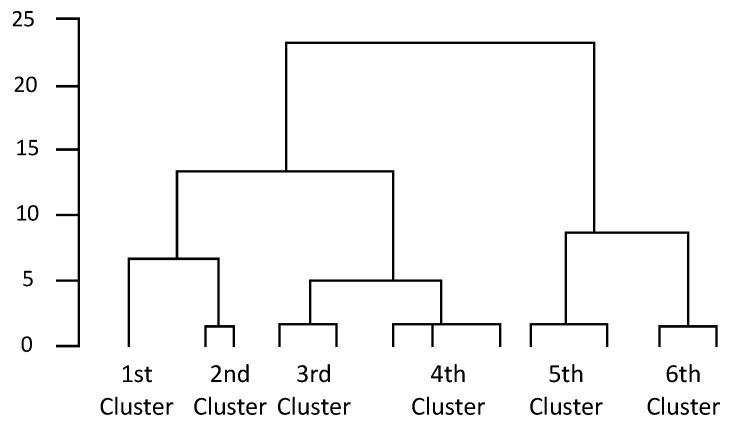
Cluster dendrogram.

Cluster analysis with Ward’s method was performed using the scores of eight items each for “self-susceptibility,” “child-susceptibility,” “economic impact of self-susceptibility (self-seriousness),” and “economic impact of child-susceptibility (child-seriousness)” for cancer and COVID-19; six clusters were obtained (Figure 1). There were 227, 299, 58, 117, 254, and 302 respondents in the first, second, third, fourth, fifth, and sixth clusters, respectively. A Chi-square statistic was conducted to examine the bias in the headcount ratio; the result was significant (*χ*^2^ = 240.389, *df* = 5, *p* < 0.001).

Next, an analysis of variance—using the six clusters as independent variables and the eight items of self-susceptibility, child-susceptibility, self-seriousness, and child-seriousness as dependent variables—was conducted. Significant group differences were found for all eight items: self-susceptibility toward “cancer” (F [5, 1256] =100.763, *p* < 0.001), child-susceptibility toward “cancer” (F [5, 1256] = 119.352, *p* < 0.001), self-seriousness toward “cancer” (F [5, 1256] = 374.079, *p* < 0.001), child-seriousness toward “cancer” (F [5, 1256] = 381.571, *p* < 0.001), self-susceptibility toward “COVID-19” (F [5, 1256] = 343.798, *p* < 0.001), child-susceptibility toward “COVID-19” (F [5, 1256] = 323.701, *p* < 0.001), self-seriousness toward “COVID-19” (F [5, 1256] = 430.107, *p* < 0.001), and child-seriousness toward “COVID-19” (F [5, 1256] =424.536, *p* < 0.001). Results of multiple comparisons by Tukey’s honestly significant difference method (5% level) are given in Table 4.

**Figure 2:**
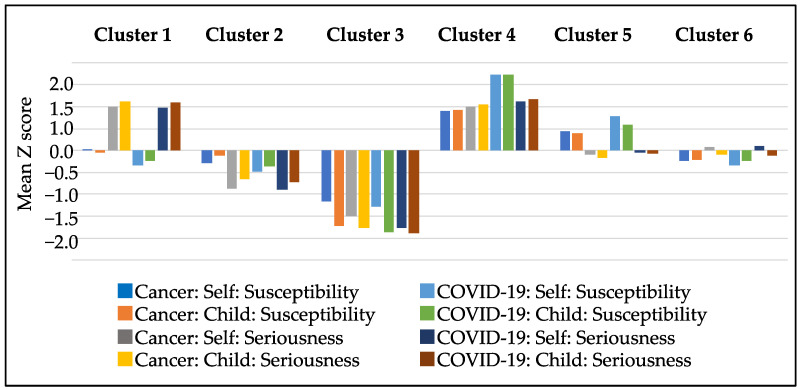
Clustered bar graph by susceptibility and seriousness of cancer and COVID-19.

Based on the characteristics of each group, the six groups were named as follows: The first cluster was designated the “high seriousness group” because it was concerned only with the seriousness (economic impact) of cancer and COVID-19. The second cluster was named the “low seriousness group” because no particular concern was expressed about perceived seriousness. The third cluster was entitled the “indifferent group” because no concern was shown for either morbidity or severity. The fourth cluster was called the “high general anxiety group” because both morbidity and severity were generally high. The fifth cluster was classified the “high susceptibility group” because they were only concerned about morbidity. The sixth cluster was labeled the “moderate group” because it was intermediate and did not fall into either of the two categories. A clustered bar graph is shown in Figure 2. It compares the mean of each categories separated by type of disease (cancer or COVID-19 infection), target (child or self), and threat (susceptibility and seriousness).

### 3.4. Logistic Regression Analysis

This study sequentially introduced groups of variables—including demographic variables, perceived threat variables, sociopsychological variables, cues to action variables, and benefits and barrier variables—into the HBM. Hence, we conducted logistic regression analysis for this model to calculate the adjusted odds ratio (OR)—with 95% confidence interval (CI)—for the intention of HPV vaccination. The logistic regression model was performed with adjustments for all potential factors as listed in Table 1. The intention to vaccinate against HPV in the presence of common adverse reactions was used as an explanatory variable. Attributes such as knowledge about COVID-19 and HPV, media contact, discussion between partners, discussion between parent and child, psychological factors, intention of COVID-19 vaccination, cancer screening, advantages and disadvantages of vaccination or cancer screening, and six types of perceived threat categorized by cluster analysis were added as adjustment variables. First, attribute items were inserted into the first block as adjustment variables using the forced imputation method; then, the abovementioned variables were inserted into the following blocks (in order) using the maximum likelihood method, and finally, the sixth cluster (moderate group) classified by cluster analysis was added to the last block using the forced imputation method as contrasting indicators. The results of all blocks are shown in Table 5.

The results showed that the following 14 explanatory variables were significant, out of which 8 were associated with an improved intention to vaccinate against cervical cancer: sources of information used to make COVID-19 judgments—family doctor’s advice (OR = 1.57, CI = 1.06–2.32, *p* = 0.02), information from reputable social networking sites (SNS) (OR = 2.40, CI = 1.33–4.33, *p* = 0.00); realization that COVID-19 may spread even before symptoms such as cough and sore throat appear (OR = 1.16, CI = 1.00–1.34, *p* = 0.05); awareness of media reports about the development of a vaccine to prevent COVID-19 (OR = 4.09, CI = 2.08–8.06, *p* = 0.00); importance of HPV prevention by vaccination (OR =1.65, CI = 1.30–2.10, *p* = 0.00); intention to be vaccinated against COVID-19, “if there are common side effects, I will get myself vaccinated against COVID-19” (OR = 1.97, 1.29–3.00, *p* = 0.00), and “if there are common side effects, I will get my child vaccinated against COVID-19” (OR =4.16, CI = 2.79–6.19, *p* = 0.00); gain (benefit) from HPV vaccination (OR = 1.06, CI = 1.02–1.12, *p* = 0.01). However, six other factors were associated with a decrease in intention: information from reputable SNS as credible information (OR = 0.41, CI = 0.21–0.81 *p* = 0.01); awareness of media reports about adverse reactions to vaccines that prevent cervical cancer (OR = 0.59, CI = 0.41–0.86, *p* = 0.01), “not vaccinate my child to prevent cervical cancer” (dummy) (OR = 0.45, CI = 0.28–0.72, *p* = 0.00); “not decide to vaccinate my child to prevent cervical cancer” (dummy) (OR = 0.59, CI = 0.41–0.85, *p* = 0.01); loss (barrier) from cervical cancer screening (OR = 0.95, CI = 0.91–0.99, *p* = 0.01); perceived threat of “group with low seriousness” (OR = 0.58, CI = 0.37–0.93, *p* = 0.02). The model fit was 80.5% of the total.

## 4. Discussion

**Figure 3:**
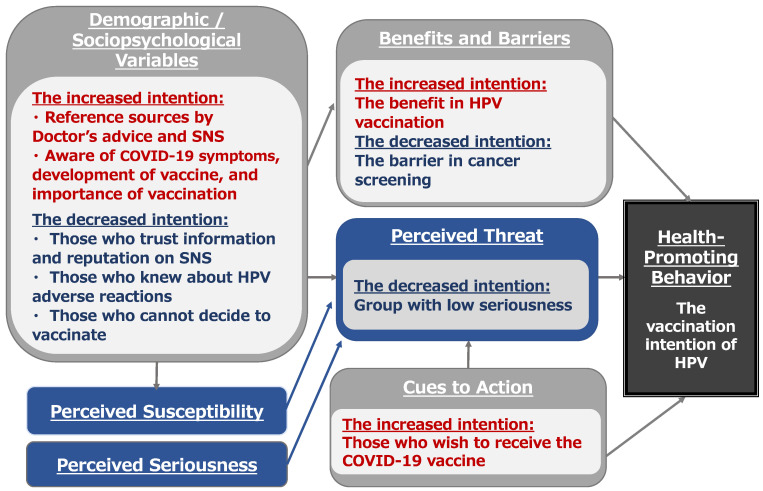
HBM variables could potentially affect people’s acceptance of receiving an HPV vaccine. Note: Sociopsychological Variables includes health information and perception, psychological factors such as psychological reactance, perceived vulnerability to disease, and neurogenic tendencies, and discussion such as communication with husband and wife or parent and child. Benefits and Barriers imply advantages and disadvantages on vaccinations and screening behaviors. Cues to Action include the intention toward COVID-19 vaccination and the availability of cancer screening.

The main purpose of this study was (1) to classify the patterns of threat perception toward cancer and COVID-19 and (2) to examine whether HBM variables, especially cues to action, as the intention for COVID-19 vaccination can explain the intention to vaccinate against cervical cancer. Figure 3 shows that the HBM variables could potentially influence the intention to get the HPV vaccine.

### 4.1. Perceived Threat

Even though the clusters were classified according to the combination of susceptibility to—and seriousness of—each disease, there are few differences in perceived threats between cancer and COVID-19. The cumulative incidence of cancers in Japanese people under 75 years of age was 32.5% for all body parts [2]. Although the infection rate of COVID-19 is high, the rates of severe disease and mortality are not as high as those of cancer. However, the results show that the perceived threat from COVID-19 and cancer does not significantly change, which shows that the COVID-19 infection is considered to be as much of a threat as cancer. One possible explanation of this might be social media’s direct involvement with COVID-19 patients or constant contact with medical information. Furthermore, the loss of life from cervical cancer caused by HPV infection is years to decades away and is discounted compared to death from COVID-19 infection, which occurs right in front of our eyes. For these reasons, people worried about COVID-19 infections are also worried about contracting cancer, but not vice versa.

The results of the logistic regression analysis of the perceived threats showed that while those with lower perceived seriousness were more likely to have lower HPV vaccination intention, and that other perceived risk (segmentation) of contracting cancers or COVID-19 did not shape HPV vaccination intention. Our study cannot mention that there is clearly no evidence of an association between perceived threats and HPV vaccination intention; however, possible reasons for the lack of a specific relationship between most of perceived threats and HPV vaccination intention are discussed below. First, approximately 60% of the subjects wanted to be vaccinated against HPV and COVID-19, which seems to indicate a large subject bias. Second, the lack of significant differences between cancer and COVID-19 in terms of susceptibility and severity in the results of the cluster analysis could also be a major factor differentiating those relationships. Finally, regardless of the severity or morbidity of the disease, when people are exposed to COVID-19 information through the media, they may experience a desire to vaccinate and thus protect themselves. Several Japanese people avoided HPV vaccination due to media influence; meanwhile, daily COVID-19 reports have strengthened the overall awareness of vaccination. For these reasons, no clear relationship could be derived between risk perception and HPV vaccination intention.

### 4.2. Cues to Action

The results of the logistic regression analysis of the HPV vaccination intention showed that, as hypothesized, the high intention to vaccinate against COVID-19 was significantly associated with the intention to vaccinate against cervical cancer. This might be because repeated daily reports of COVID-19 infections and restrictions on behavior have made people feel more threatened by infectious diseases. In addition, since the government is encouraging health management within organizations, such as companies and schools, the increasing interest in health has created a more conducive environment for vaccination. Furthermore, as of November 2020, although no COVID-19 vaccine had been developed or inoculated, the prevalence of infections in Japan and worldwide had been reported extensively in the media, which may have induced a sense of anxiety and thus stimulated health awareness. Likewise, the Japan 2020 Summer Olympics were postponed, and the government supplied more COVID-19-related information. Under these circumstances, 65.7% of the respondents wanted their children to be vaccinated against COVID-19. With this improvement in health awareness, it is likely that those who want their children to receive the new COVID-19 vaccine also want them to receive the HPV vaccine.

### 4.3. Sociopsychological Factors

Furthermore, people who consult family doctors and SNS to seek reliable information about COVID-19 tend to have an increased intention toward vaccination. This is because they trusted the opinions of their family physicians and other medical specialists even before the COVID-19. This could imply that people had pre-existing knowledge about the effectiveness of HPV vaccine because of their doctors’ recommendations. Regarding SNS, due to the overwhelming quantity of information generated by consumer-generated media, which refers to any written or audio contents created by end users, has acquired the potential to shape collective knowledge and become an important social influencer, comparable to television and newspapers [15]. In Japan, after the Great East Japan Earthquake, SNS such as Twitter and LINE were used as communication tools and played an active role in confirming the safety of people in areas where the government was unable to do so [16]. Similarly, in the current situation, no one knows about the actual conditions of—or exact solutions to—COVID-19. Therefore, it is likely that information from doctors and SNS became a basis for decision making and influenced vaccination intentions. Additionally, we found that those aware of COVID-19 symptoms, COVID-19 vaccine development, and the importance of the HPV vaccination have more likelihood of willingness to get the HPV vaccination. Greater contact with SNS and medical professionals will increase awareness about disease information and the importance of vaccines. Accordingly, information from doctors and SNS can predispose people to realize the effectiveness of HPV vaccination.

Additionally, people who are unable to decide whether to vaccinate their children for cervical cancer are apt to exhibit decreased vaccination intention. Although their reasons for indecision are unclear, present bias seems to play a role. Decision making about medical care and health involves comparisons between the benefits of future health and current loss because of financial burden and the pain of treatment, in which an important role is played by the behavioral economics of time preferences. People who tend to discount future benefits and have a powerful procrastination tendency due to present bias may be less likely to engage in aggressive medical and healthcare behaviors [17,18]. For example, the benefits of improved health or prevention of worsening disease through medical examinations or medication are more likely to be felt in the future. In such cases, future benefits are significantly discounted, and present costs are perceived to be large. As a result, health-related behavior is not seriously engaged in, and consequently, important medical decisions are delayed. Present bias also reduces people’s likelihood to undergo screening tests and to take the influenza vaccine [19]. Therefore, people who are unable to decide may also be affected by present bias, which may lead to postponed decision-making, and thus decrease vaccination intention.

### 4.4. Benefits and Barriers

The greater the knowledge about HPV vaccination benefits, the higher the intentions to be vaccinated against HPV. The five major benefits of HPV vaccination are as follows: prevention of cervical cancer in children (increase life expectancy), reduction in the number of worries (reduce the psychological burden), less disruption of the child’s daily life (work and future family) in the future, reduction in economic burden, and less isolation from the surrounding inoculation situation. These benefits mainly include protecting themselves and their children from physical and sociopsychological factors in the future.

In contrast, those aware of the adverse reaction of HPV vaccination and have a negative attitude toward cancer screening are likely to exhibit decreased vaccination intention. The five barriers to cancer screening are as follows: visiting a doctor will force them to face their disease-related anxiety; the screening tests are physically demanding; children are discouraged from receiving treatment because continual treatment is required; a lot of time is spent on treatment after cancer is diagnosed, thus affecting their planning for the future; children dislike screening. These items were composed based on physical and psychological risks, seemingly related to anticipatory anxiety and immediate concerns. In other words, it is highly likely that they have exhibit “present bias” and “loss aversion,” undesirable decision-making tendencies to chase trivial immediate gains (immediate rewards) rather than large future gains (delayed rewards). Further, some studies demonstrated that risk-averse individuals are less likely to undergo breast cancer screening to avoid the risk from action [20,21]. While breast cancer screening lowers the risk of delayed cancer detection, treatment may still fail, which indicates that screening does not eliminate the risks. Consequently, in situations where risks are involved, risk-averse individuals may not engage in proactive medical and health behaviors; the results of this study reflect this situation.

### 4.5. Limitations and Future Prospects

Vaccination and early medical examination are important for cervical cancer prevention. In this study, we found that the intention of HPV vaccination increased with the growth of health consciousness owing to the COVID-19 outbreak. This result generates important data for improving the HPV vaccination rate in Japan in the future. Further interventional studies—for example, wherein HPV and COVID-19 vaccination are simultaneously recommended—will be necessary in the future.

In contrast, the present results did not specify the relationship between all risk perceptions and vaccination intention in the HBM. The probability of adverse health effects in the form of risk assessment is important; however, this study failed to provide sufficient assessment to our model. Therefore, it is necessary to conduct further analysis by setting up a model for indirect influences, such as the intention to be vaccinated against new COVID-19 infections. In the future, we will also explore the tendency of present bias and avoidance risk. We can expect to see behavioral changes by making present or future benefits larger or present costs smaller. It has also been reported that HPV vaccination rates in some regions have increased (from 0.6% as of 2016 to 23.4% in 2020); this can be attributed to awareness-raising videos for young people by VTubers (virtual YouTuber) on YouTube and the use of these videos to raise awareness in schools [22]. We believe that further research that considers the influence of these SNS and other contributory factors is needed.

Furthermore, different communities in each region may have different perceptions of vaccines and their impact on vaccination intentions. Just as different urban and rural communities have different attitudes to infectious diseases, it is expected that their perceptions of vaccines will also differ. However, regional differences were not considered in this study. Therefore, future research should take these geographical divides into account.

In addition, since this study deals with the intention to inoculate, we consider it necessary to continue this project and include the impact on actual inoculation and consultation behavior. As of January 13, 2022, the COVID-19 vaccine has been developed, and the vaccination rate for the COVID-19 vaccine in Japan is as high as 79.0%, which is high compared to other countries. Moreover, it has been decided that the active recommendation of HPV vaccination be resumed in April 2022. With these changes in health awareness, we need to conduct additional research to determine whether the HPV vaccination intention has increased and whether there has been a change in the actual vaccination rate.

Simultaneously, immunization stress-related responses (ISRRs), which appear after vaccination, cannot be overlooked. A certain number of adverse events have been reported as nocebo effects, which are not caused by the vaccine component, after vaccination. These nocebo effect reactions are caused by vaccination-related anxiety, and in 2019, the WHO proposed the concept of ISRRs [23]. ISRRs are reported to be more likely to occur when new immunizations are introduced and when changes occur in the routine vaccination program. Healthcare professionals need to be aware of ISRRs and respond quickly before these adverse reactions spread rapidly through the media and social media. Therefore, it will be necessary to incorporate the ISRRs perspective into cervical cancer vaccine awareness-raising activities in the future.

## 5. Conclusions

The HBM could partially predict each factor influencing people getting vaccinated against HPV. Our findings indicate that the intention of COVID-19 vaccination increases the likelihood of getting HPV vaccination. This study recognized the reliance on mass media (SNS) and medical doctors for COVID-19 information and considered these to be reliable sources. In contrast, those who trust SNS information and are aware of news reportage on cervical cancer and adverse reactions to it, are less likely to get the HPV vaccination. However, even though only 3% of the total female participants were vaccinated against cervical cancer, 65.7% wanted their children to be vaccinated against COVID-19, while 67.7% also wanted the HPV vaccination for themselves. This is probably because repeated daily reports of COVID-19 infections and restrictions have made people more health conscious and aware of the danger of infectious diseases. Although the bias of the subjects should be considered, this result is considered to be important data for improving the HPV vaccination rate in Japan in the future. However, the extent to which this vaccination intention is linked to actual health behaviors is not clear from the present study. Therefore, further research on actual health behaviors and further intervention studies, such as recommending simultaneous HPV and COVID-19 vaccination, will be necessary in the future.

## Figures and Tables

**Table 1:** Main Survey Items.

1. Attributes
Gender, Age, Severe medical history, Geographic Location, Occupation, Age of daughter
2. Perceived seriousness and perceived susceptibility toward cancer and COVID-19
16-item 7-point scale (Likert scale recoded to “strongly disagree”, “strongly agree,” and “neither strongly agree nor strongly disagree”) that asks about the subjective probability of illness for oneself and one’s children (breast cancer, cervical cancer, colorectal cancer, and COVID-19 infection for women; lung cancer, stomach cancer, colorectal cancer, and COVID-19 infection for men) and the economic impact of each of the four illnesses on participants or their children
3. Literacy and beliefs about COVID-19, HPV vaccines, and cervical cancer screening
16-item 6-point scale (Likert scale recoded to “strongly disagree”, “strongly agree”) that assesses knowledge regarding cervical cancer, such as “cervical cancer is mainly transmitted through sexual intercourse” and beliefs such as “I think it is important to prevent COVID-19 by vaccination”4-item, 2-point scale (yes or no) about whether the respondent knows some news about vaccines, whether the respondent knows about adverse reactions toward HPV vaccines, and whether the vaccine is reliable and feasible
4. Experience of cancer screening
1-item 5-point scale (1. Examined within the last one year, 2. Examined within the last two years, 3. Examined more than two years ago, 4. Not ever, 5. Do not know)
5. Intention of vaccination
6-item 6-point scale (Likert scale recoded to “strongly disagree” and “strongly agree”) about whether the respondents vaccinate or allow their child to be vaccinated in varied vaccination situations (no risk of side effects, common side effects, unknown/serious side effects) in the case of the COVID-19 vaccine. A 3-item, 6-point scale (Likert scale recoded to “strongly disagree”, “strongly agree”) that evaluates whether the respondents vaccinate or allow their child to be vaccinated in the aforementioned circumstances, in the case of the HPV vaccine
6. Discussion between husband and wife / parent and child
4-item 2-point scale (yes or no) on whether there is discussion about the HPV and COVID-19 vaccines
7. Benefit/barrier
30-item 7-point scale (Likert scale recoded to “strongly disagree,” “strongly agree,” and “neither strongly agree nor strongly disagree”); 5 items—each about benefits and barriers on two types of vaccination (COVID-19 and HPV) and screening behavior (cervical cancer)
8. Health literacy and mass media
Health literacy (9-item 6-point scale: Likert scale recoded to “strongly disagree” and “strongly agree”), means of getting health information (12-item 2-point scale: yes or no), reference sources for judging COVID-19 (12-item 2-point scale), reference sources for judging cervical cancer (12-item 2-point scale: yes or no), trusting information (12-item 2-point scale), method of sharing trusting information (7-item, 2-point scale: yes or no), method of checking suspicious information (1-item 6-point scale: “All Search” and ”Never search”), frequency of checking health information (1-item 6-point scale: ”Several times a day” and “Hardly ever”)
9. Psychological characteristics
The Hong Psychological Reactance Scale (Hong & Page, 1989) (14-item 5-point scale), the Japanese version of the Perceived Vulnerability to Disease Scale (Fukukawa et al., 2014) (15-item 5-point scale), and the neurogenic tendencies of the Big Five (Namikawa et al., 2012) (5-item 5-point scale)

**Table 2:** Participant characteristics.

Total *n* = 1257		*n*	%
Age		*M* = 45.42; *SD* = 5.83	
	20–29	1	0.1
	30–39	196	15.6
	40–49	774	61.6
	50–59	268	21.3
	60–69	18	1.4
Gender			
	Male	423	33.7
	Female	834	66.3
Marital Status			
	Yes	1180	93.9
	No	77	6.1
Experience of cancer screening test			
	Yes	830	66.0
	No	324	25.9
	Not remember	103	8.2
Experience of flu vaccine			
	Yes	1028	81.8
	No	192	15.3
	Not remember	37	2.9
Experience of HPV vaccine			
	Yes	26/834	3.1
	No	770/834	92.3
	Not remember	38/834	4.6
Willing to give children new COVID-19 vaccine with common side effect			
	Yes	827	65.7
	No	430	34.2
Willing to give children HPV vaccine with common side effect			
	Yes	852	67.7
	No	405	32.2

**Table 3:** Correlation of susceptibility and seriousness toward cancer and COVID-19.

		1.	2.	3.	4.	5.	6.	7.	8.
Cancer	1. Self + Susceptibility	1.000							
	2. Child + Susceptibility	0.562 **	1.000						
	3. Self + Seriousness	0.286 **	0.257 **	1.000					
	4. Child + Seriousness	0.253 **	0.333 **	0.741 **	1.000				
COVID-19	5. Self + Susceptibility	0.487 **	0.376 **	0.258 **	0.228 **	1.000			
	6. Child + Susceptibility	0.360 **	0.506 **	0.293 **	0.312 **	0.729 **	1.000		
	7. Self + Seriousness	0.254 **	0.244 **	0.756 **	0.595 **	0.298 **	0.367 **	1.000	
	8. Child + Seriousness	0.261 **	0.295 **	0.626 **	0.758 **	0.286 **	0.372 **	0.737 **	1.000

** The correlation coefficient is significant (two-sided) at the 1% level.

**Table 4:** Six clusters of cancer and COVID-19 (mean, *SD*, and multiple comparison for each group).

	Cluster 1	Cluster 2	Cluster 3	Cluster 4	Cluster 5	Cluster 6	F-Value	
Cancer								
Self-susceptibility	0.0143	−0.2798	−1.1654	0.8975	0.4412	−0.2286	100.763	***
4 > 5 > 1 > 6, 2 > 3	(0.745)	(0.501)	(1.133)	(0.952)	(0.694)	(0.705)		
Child susceptibility	−0.0505	−0.1084	−1.7305	0.933	0.3832	−0.2061	119.352	***
4 > 5 > 1, 2, 6 > 3	(0.701)	(0.410)	(1.136)	(1.012)	(0.684)	(0.854)		
Self-seriousness	1.0035	−0.8678	−1.4893	1.0023	−0.0851	0.0743	374.079	***
1, 4 > 6, 5 > 2, 3	(0.543)	(0.457)	(1.175)	(0.559)	(0.625)	(0.652)		
Child seriousness	1.1072	−0.6623	−1.7752	1.0467	−0.1641	−0.1031	381.571	***
1, 4 > 6, 5 > 2 > 3	(0.583)	(0.388)	(1.128)	(0.593)	(0.594)	(0.717)		
COVID-19								
Self-susceptibility	−0.3455	−0.4898	−1.2789	1.7198	0.7779	−0.3303	343.798	***
4 > 5 > 6, 1, 2 > 3	(0.734)	(0.472)	(1.232)	(0.832)	(0.683)	(0.430)		
Child susceptibility	−0.2463	−0.3636	−1.8768	1.7222	0.5776	−0.2474	323.701	***
4 > 5 > 1, 6, 2 > 3	(0.763)	(0.371)	(1.186)	(0.892)	(0.738)	(0.463)		
Self-seriousness	0.9748	−0.9064	−1.7749	1.1142	−0.0431	0.1101	430.107	***
4, 1 > 6, 5 > 2 > 3	(0.587)	(0.432)	(0.965)	(0.479)	(0.709)	(0.635)		
Child seriousness	1.0839	−0.7162	−1.8881	1.1564	−0.0749	−0.1281	424.536	***
4, 1 > 6, 5 > 2, 3	(0.560)	(0.456)	(0.827)	(0.558)	(0.698)	(0.668)		

*** *p* < 0.001.

**Table 5:** Logistic regression analysis of common adverse reactions to HPV vaccination intention.

Block	Explanatory Variables	B	(SE)	Wald	*p*-Value	Odds-Rate	95% Confidence Interval
Demographic Variables	Gender	−0.309	0.265	1.365	0.243	0.734	0.437–1.233
	Age	0.006	0.016	0.151	0.698	1.006	0.975–1.039
	Severe medical history	−0.064	0.295	0.047	0.828	0.938	0.526–1.673
	Geographic Location	−0.002	0.007	0.074	0.785	0.998	0.984–1.013
	Occupation	−0.002	0.023	0.011	0.916	0.998	0.953–1.044
	Age of daughter	−0.043	0.060	0.502	0.479	0.958	0.852–1.078
Health literacy, Media contact (Sociopsychological Variables)	Health Literacy: HPV vaccine can reduce cervical cancer deaths	0.183	0.118	2.401	0.121	1.201	0.953–1.513
	Health Literacy: Even with the common adverse reactions to COVID-19 vaccine, I would still get vaccinated	−0.005	0.090	0.003	0.956	0.995	0.835–1.186
	Reference source for COVID-19 judgments: Notifications from local governments and health centers	0.300	0.174	2.977	0.084	1.350	0.960–1.898
	Reference source for COVID-19 judgments: Advice from your family doctor or other medical professionals	0.450	0.199	5.113	0.024	1.569	1.062–2.318
	Reference source for COVID-19 judgement: Information and reputation on social networking service (SNS) *	0.874	0.302	8.391	0.004	2.397	1.327–4.332
	Reference sources for HPV judgement: Blogs and experiences from other people.	0.360	0.321	1.259	0.262	1.433	0.764–2.689
	Information sources to trust: Information and reputation on SNS	−0.888	0.343	6.697	0.010	0.411	0.210–0.806
	How to share credit information: Blogs and other posts on the Internet except SNS	−1.238	0.587	4.442	0.035	0.290	0.092–0.917
Perceptions and beliefs about COVID-19 and cervical cancer (Sociopsychological variables)	Recognition of COVID-19: Infection may spread even before symptoms such as cough and sore throat appear.	0.147	0.074	3.959	0.047	1.158	1.002–1.338
	Awareness of media reports about the development of a preventive vaccine for the COVID-19	1.409	0.346	16.604	0.000	4.093	2.078–8.060
	Vaccines against new COVID-19 are reliable	−0.041	0.104	0.156	0.692	0.960	0.783–1.176
	Aware of news reports about adverse reactions to the cervical cancer vaccine	−0.527	0.189	7.806	0.005	0.591	0.408–0.855
	Not vaccinate my child to prevent cervical cancer (dummy)	−0.806	0.241	11.206	0.001	0.447	0.279–0.716
	Not decide to vaccinate my child to prevent cervical cancer (dummy)	−0.533	0.189	7.927	0.005	0.587	0.405–0.851
	Importance of HPV prevention through vaccination	0.503	0.122	17.084	0.000	1.654	1.303–2.100
Intention of COVID-19 vaccination (Cues to action)	If there are general side effects, I will vaccinate myself against COVID-19 (dummy)	0.676	0.215	9.856	0.002	1.965	1.289–2.996
	If there are general side effects, I will vaccinate my child against COVID-19 (dummy)	1.425	0.203	49.222	0.000	4.160	2.793–6.194
Advantages and disadvantages in vaccination and screening (Benefits and barriers)	Advantages from HPV vaccination	0.062	0.024	6.748	0.009	1.064	1.015–1.115
	Disadvantages from getting cervical cancer screening	−0.053	0.022	6.099	0.014	0.948	0.909–0.989
Clusters (Perceived Threat)	Moderate group			6.203	0.287		
	High seriousness group	−0.375	0.276	1.850	0.174	0.687	0.400–1.180
	Low seriousness group	−0.539	0.238	5.128	0.024	0.583	0.366–0.930
	Indifferent group	−0.414	0.398	1.080	0.299	0.661	0.303–1.443
	High general anxiety group	−0.173	0.384	0.204	0.652	0.841	0.396–1.785
	High susceptibility group	−0.486	0.249	3.800	0.051	0.615	0.377–1.003
chi-square goodness-of-fit test (Hosmer and Lemeshow-test)		4.60 (*p* = 0.80)					

Note: Forced imputation method for demographic variables and clusters, maximum likelihood ratio method for others. For perceived threat, the sixth cluster classified by cluster analysis was used as a contrast index. * Social networking service (SNS) is an online vehicle for “creating relationships with other people”, and it differs from social media which include all Internet sources.

## Data Availability

Data supporting the results of this study are available upon request from the authors.

## Supplementary Materials

The following supporting information can be downloaded at: https://www.mdpi.com/article/10.3390/vaccines10050829/s1, Table S1: All questions in English [24,25,26].
