# Supplementary material for: Impact of the Threat of COVID-19 Infections on the Perceived Risk to HPV Vaccination
Source: Vaccines (Basel). 2022 May 23;10(5):829. doi: 10.3390/vaccines10050829 (PMC9147999; doi:10.3390/vaccines10050829)
Supplement: Supplementary file 1 [file vaccines-10-00829-s001.zip › vaccines-1722094-supplementary.pdf]

Supplementary Table S1

All questions in English

For women

1.	あなたの性別と年齢を教えてください。<br>What is your gender and age?
2.	あなたの家族構成についてお伺いします。<br>What is your family structure?
3.	あなたが今まで接種したことのあるワクチンを教えてください。<br>Please tell us which vaccines you have been vaccinated against.
	(インフルエンザ、子宮頸がん（HPV)、三種(百日咳・ジフテリア・破傷風)混合(DPT)、日本脳炎、その他のワクチン）<br>(Influenza, cervical cancer (HPV), triad combined (diphtheria / pertussis / tetanus) (DPT), Japanese encephalitis, and other vaccines)
4.	あなたは以前がんなどの病気に罹患した経験がありますか？ある場合、その病名を教えてください。<br>Have you ever had cancer or any other disease before? If yes, what is the name of the disease?
5.	一生涯のうち一般的な日本人が以下の病気にかかる可能性はどれくらいだと思いますか？それぞれの項目に、最も当てはまるものをお答えください。（7 件法）

	How likely do you think it is that the average Japanese person will suffer from the following diseases during their lifetime? For each item, please answer the most applicable. (7-point scale)
	(子宮頸がん、乳がん、大腸がん、新型コロナウイルス感染症、インフルエンザ)<br>(cervical cancer, breast cancer, colorectal cancer, new coronavirus infection, influenza)
6.	一生涯のうち自分自身が以下の病気にかかる可能性はどれくらいだと思いますか？それぞれの項目に、最も当てはまるものをお答えください。（7 件法）<br>How likely do you think you are to get any of the following diseases yourself in your lifetime? For each item, please answer the most applicable. (7-point scale)
	(子宮頸がん、乳がん、大腸がん、新型コロナウイルス感染症、インフルエンザ)<br>(cervical cancer, breast cancer, colorectal cancer, new coronavirus infection, influenza)
7.	一生涯のうちあなたの子どもが以下の病気にかかる可能性はどれくらいだと思いますか？それぞれの項目に、最も当てはまるものをお答えください。（7 件法）

	How likely do you think your child is to get any of the following diseases during his or her lifetime? For each item, please answer the most applicable. (7-point scale)
	(子宮頸がん、乳がん、大腸がん、新型コロナウイルス感染症、インフルエンザ)<br>(cervical cancer, breast cancer, colorectal cancer, new coronavirus infection, influenza)
8.	将来あなたが以下の病気にかかることによる経済的負担や仕事などへの影響はどれくらいだと思われますか？それぞれの項目に、最も当てはまるものをお答えください。（7 件法）<br>How much do you think the financial burden and impact on your work would be if you were to suffer from any of the following diseases in the future? For each item, please answer the most applicable.(7-point scale)
	(子宮頸がん、乳がん、大腸がん、新型コロナウイルス感染症、インフルエンザ)<br>(cervical cancer, breast cancer, colorectal cancer, new coronavirus infection, influenza)
9.	将来あなたの子どもが以下の病気にかかることによる経済的負担や仕事などへの影響はどれくらいだと思われますか？それぞれの項目に、最も当てはまるものをお答えください。（7 件法）<br>How much do you think the financial burden and impact on your work would be if your child were to suffer from any of the following diseases

	in the future? For each item, please answer the most applicable. (7-point scale)
	(子宮頸がん、乳がん、大腸がん、新型コロナウイルス感染症、インフルエンザ)<br>(cervical cancer, breast cancer, colorectal cancer, new coronavirus infection, influenza)
10.	あなたは新型コロナウイルスについて、以下の情報を知っていますか。それぞれの項目に、最も当てはまるものをお答えください。（6 件法）<br>Do you know about the following information on COVID-19? For each item, please answer the one that best applies. (6-point scale)
	・コロナウイルスは遺伝情報として RNA ウイルスの一種（一本鎖 RNA ウイルス）である<br>Coronaviruses are a type of RNA virus (single-stranded RNA virus) with genetic information
	・一般的には飛沫感染、接触感染で感染する<br>Generally transmitted by droplet or contact infection.
	・咳や咽頭通などの症状が明らかになる前から、感染が広がるおそれがあると言われている<br>It is said that the infection may spread even before symptoms such as coughing and pharyngeal discharge become apparent.

	・マスク着用、手洗いやアルコールによる消毒などが感染の予防に重要である<br>The use of masks, hand washing and disinfection with alcohol are important to prevent infection.
	・感染の有無を調べるには PCR 検査などがある<br>PCR tests and other tests are available to determine the presence of infection.
11.	新型コロナウイルス予防ワクチンの開発についての報道をご存知ですか？（2 件法）<br>Are you aware of any media reports about the development of a vaccine to prevent new coronaviruses? (2-point scale)
12.	新型コロナウイルス予防ワクチンの開発は成功し、実施されるようになると思いますか？（2 件法）<br>Do you think the development of a vaccine to prevent COVID-19 will be successful and become implemented? (2-point scale)
13.	新型コロナウイルスの予防など新型コロナウイルス対策のワクチンは信頼できると思いますか？（6 件法）<br>Do you think that vaccine against COVID-19 is reliable? (6-point scale)
14.	以下のような条件下で、あなたは新型コロナウイルスを予防するためのワクチンを接種しようと思いますか？（6 件法）

	Under the following conditions, would you be willing to be vaccinated to prevent COVID-19? (6-point scale)
	副作用などリスクがまったくない場合<br>If there are no side effects or other risks at all.
	痛みやアレルギー反応などインフルエンザワクチンなどすでに普及しているワクチンと同程度の副作用のリスクがある場合<br>If there is a risk of side-effects comparable to those of vaccines already in widespread use, such as influenza vaccines, including pain and allergic reactions.
	まだ明らかになっていない副作用などのリスクがある場合<br>If there is a risk of side-effects or other risks that have not yet been identified.
15.	以下のような条件下で、あなたは新型コロナウイルスを予防するためのワクチンを「子ども」に接種させると思いますか？（6 件法）<br>Under the following conditions, would you allow your **child** to be vaccinated against COVID-19? (6-point scale)
	副作用などリスクがまったくない場合<br>If there are no side effects or other risks at all.
	痛みやアレルギー反応などインフルエンザワクチンなどすでに普及しているワクチンと同程度の副作用のリスクがある場合<br>If there is a risk of side-effects comparable to those of vaccines already in widespread use, such as influenza vaccines, including pain and allergic reactions.

	まだ明らかになっていない副作用などのリスクがある場合<br>If there is a risk of side-effects or other risks that have not yet been identified.
16.	有効な治療法が開発されたとしても、ワクチンなどを使って新型コロナウイルスを予防することは大事だと思いますか？（6 件法）<br>Even if an effective treatment is developed, do you think it is important to prevent COVID-19 through vaccines or other means? (6-point scale)
17.	配偶者（パートナー）がいる方に質問します。子どもへの新型コロナウイルスのワクチン接種について配偶者と検討したことがある、あるいは、する予定はありますか？（2 件法）<br>Question for those with a spouse (partner). Have you considered or do you plan to consider vaccinating your children against COVID-19 with your spouse? (2-point scale)
18.	問 17 で「いいえ」を選択した方に伺います。「いいえ」を選択した理由を以下から選択してください。<br>If you selected “No” in Q17. Please select the reason why you chose “No” from the following options.<br>・そこまで重要だと考えていないから<br>I don't think it is that important.<br>・子どもの選択であるから<br>It is my child's choice.

	・相談してもしょうがないから<br><br>There's no point in discussing it.<br><br>・情報共有のみで良いと思うから<br><br>I think it's good to just share information.<br><br>・相談しても聞いてもらえないだろうから<br><br>My partner wouldn't talk to me about it and wouldn't listen to me.<br><br>・その他<br><br>Other.
19.	新型コロナウイルスのワクチン接種について子どもと話し合ったことがありますか？（2 件法）<br><br>Have you ever discussed vaccination against the new coronavirus with your child? (2-point scale)
20.	問 19 で「いいえ」を選択した方に伺います。「いいえ」を選択した理由を以下から選択してください。<br><br>If you selected “No” in Q19. Please select the reason why you chose “No” from the following options.<br><br>・こうした話し合いを子どもが嫌がると思うから<br><br>I think my child would not like these discussions.<br><br>・結局判断を任されるから<br><br>I'm left to make the decision in the end.<br><br>・親が意思決定すべきだから

	Parents should make decisions.<br>・関心がなさそうだから<br>My child doesn't seem interested.<br>・説明したら、接種したくないと言いそうだから<br>I don't think my child would want to be vaccinated if I explained it to her.<br>・関係がギクシャクしそうだから<br>The relationship is likely to be strained.<br>・子どもと話しにくいため<br>I am reluctant to talk to my children.<br>・その他<br>Other.
21.	子どもに新型コロナウイルスのワクチン接種を「する」ことで、どんなメリット（いいこと）があると、あなたは考えていますか。それぞれの項目に、最もあてはまるものをお答えください。（7 件法）<br>What do you think are the benefits (good things) of “vaccinating” your child against COVID-19? For each item, please answer the one that best applies. (7-point scale)
	感染を防ぐことができる<br>Able to prevent infection.
	入院や隔離を回避できる<br>Avoid hospitalisation or isolation.

	家族や職場に迷惑をかけなくて済む<br><br>No more inconvenience to family or work place.
	周りの人と同じ行動を取ることで安心できる<br><br>I feel safe in behaving in the same way as others around me.
	周囲の人に不快な思いをさせない<br><br>Not causing discomfort to those around me.
22.	子どもに新型コロナウイルスのワクチン接種を「する」ことで、どんなデメリット（悪いこと）があると、あなたは考えていますか。それぞれの項目に、最もあてはまるものをお答えください。（7 件法）<br><br>What do you think are the disadvantages (bad things) of “vaccinating” your child against COVID-19? For each item, please answer the one that applies best to you. (7-point scale)
	副反応が起こるかもしれない<br><br>Adverse reactions might occur.
	子どもに後遺症が残った場合に、自責感に苛まれる<br><br>Feeling self-condemned if my child is permanently injured.
	異常があった際に「親のせいだ」と子どもに言われるのが辛い<br><br>It is hard for children to be told that it is their parents' fault when there is an abnormality.
	問題発生時に子どもとの関係が悪くなる<br><br>Poor relationship with my child when problems occur.

	ワクチン接種しても効果がない可能性がある<br>Vaccination may not be effective.
23.	子どもに新型コロナウイルスのワクチン接種を「しない」ことで、どんなメリット（いいこと）があると、あなたは考えていますか。それぞれの項目に、最もあてはまるものをお答えください。（7 件法）<br>What do you think are the advantages (good things) of “not vaccinating” your child against the new coronavirus? For each item, please answer the one that best applies. (7-point scale)
	副反応で子どもを苦しめるリスクを回避できる<br>Avoid the risk of my child suffering from adverse reactions.
	意思決定の負担を負わずに済む<br>Not having to bear the burden of decision-making.
	子どもの人生に責任を持たずに済む<br>I don't have to take responsibility for my child's life.
	これまで通りでいられるため、ストレスが少ない<br>Less stressful as it can be business as usual.
	ワクチンによる問題が発生しないため、子どもとの関係を崩さずに維持できる<br>No problems or changes caused by the vaccine, so the relationship with my child can be maintained without disruption.

24.	子どもに新型コロナウイルスのワクチン接種を「しない」ことで、どんなデメリット（悪いこと）があると、あなたは考えていますか。それぞれの項目に、最もあてはまるものをお答えください。（7 件法）<br>What do you think are the disadvantages (bad things) of “not vaccinating” your child against the new coronavirus? For each item, please answer the one that applies best to you. (7-point scale)
	感染を防ぐことができず、感染リスクが高いままになる<br>Failure to prevent infection, leaving the patient at high risk of infection.
	入院や隔離となるリスクを抱えたままとなる<br>Remain at risk of hospitalization or quarantine.
	職場や家族に迷惑をかけるかもしれない<br>May cause problems at work or with family members.
	周りの人から白い目で見られる<br>People around you look at you in a white way.
	周囲の人に不快な思いをさせる<br>Makes people around you feel uncomfortable.
25.	あなたは子宮頸がんについて以下の情報を知っていますか？それぞれの項目に、最も当てはまるものをお答えください。（6 件法）<br>Are you aware of the following information about cervical cancer? For each item, please answer the one that best applies. (6-point scale)

	遺伝情報としてDNAウイルスの一種（環状二本鎖DNAウイルス）である<br>It is a type of DNA virus (cyclic double-stranded DNA virus) with genetic information.
	主に性交渉で感染する<br>Mainly sexually transmitted.
	感染しても症状は現れず、症状のないまま感染が広がる<br>Infection does not cause symptoms and spreads without symptoms.
	コンドームは感染予防に有効ではあるが、感染を完全に防ぐことはできない<br>Condoms are effective in preventing infection, but do not prevent infection altogether.
	感染の有無を調べるには、ハイブリッドキャプチャー法やPCR法などがある<br>Hybrid capture and PCR methods are used to test for the presence of infection.
26.	子宮頸がん予防ワクチンの副反応についての報道をご存知ですか？（2件法）<br>Are you aware of any media reports about adverse reactions to the cervical cancer prevention vaccine? (2-point scale)

27.	子宮頸がん予防ワクチンの安全性に問題があると思いますか？（6 件法）<br><br>Do you think there are safety issues with the cervical cancer prevention vaccine? (6-point scale)
28.	子宮頸がんを予防するために子どもにワクチン接種をさせると思いますか？(選択)<br><br>Would you have your child vaccinated to prevent cervical cancer? (Select one)<br><br>・勧奨が再開されなくても、接種させる<br><br>Even if the recommendation is not reintroduced, my child should be vaccinated.<br><br>・勧奨が開始されたら、接種させる<br><br>I will have my child vaccinated when the recommendation is launched.<br><br>・自分の周りや知り合いが接種してから接種させる<br><br>I would make my children vaccinate after I and others I know have been vaccinated.<br><br>・同世代の多くの子が接種していることが分かってから接種させる<br><br>I would have my child vaccinated only after I knew that many children of my generation had been vaccinated.<br><br>・接種させない<br><br>Not vaccinate.<br><br>・決められない

	I can't make up my mind.<br>・その他<br>Other
29.	以下のような条件下で、あなたは子宮頸がんを予防するためのワクチンを「子ども」に接種させると思いますか？それぞれの項目に、最も当てはまるものをお答えください。（6 件法）<br>Under the following conditions, would you allow "your child" to be vaccinated to prevent cervical cancer? For each item, please answer the one that best applies. (6-point scale)
	副作用などリスクがまったくない場合<br>If there are no side effects or other risks at all.
	痛みやアレルギー反応などインフルエンザワクチンなどすでに普及しているワクチンと同程度の副作用のリスクがある場合<br>If there is a risk of side effects comparable to those of vaccines already in widespread use, such as influenza vaccines, including pain and allergic reactions.
	重篤な副反応などのリスク（0.01%程度）がある場合<br>Risk of serious adverse reactions and other reactions (around 0.01%).
30.	ワクチンを使って、子宮頸がんを予防することは大事だと思いますか？（6 件法）<br>Do you think it is important to use vaccines to prevent cervical cancer? (6-point scale)

31.	配偶者（パートナー）がいる方に質問します。子どもへの子宮頸がん（HPV）ワクチン接種について配偶者と検討したことがある、あるいは、する予定はありますか？　（2 件法）<br>Question for those with a spouse (partner). Have you ever considered or do you plan to consider vaccinating your children against cervical cancer (HPV) with your spouse? (2-point scale)
32.	問 31 で「いいえ」を選択した方に伺います。「いいえ」を選択した理由を以下から選択してください。<br>if you selected “No” in Q31. Please select the reason why you chose “No” from the following options.<br>・そこまで重要だと考えていないから<br>I don't think it is that important.<br>・子どもの選択であるから<br>It is my child's choice.<br>・相談してもしょうがないから<br>There's no point in discussing it<br>・情報共有のみで良いと思うから<br>I think it's good to just share information.<br>・相談しても聞いてもらえないだろうから<br>My partner wouldn't talk to me about it and wouldn't listen to me.<br>・その他

	Other
33.	子宮頸がん（HPV）ワクチン接種について子どもと話し合ったことがありますか？（2 件法）<br>Have you ever discussed cervical cancer (HPV) vaccination with your child? (2-point scale)
34.	問 33 で「いいえ」を選択した方に伺います。「いいえ」を選択した理由を以下から選択してください。<br>if you selected “No” in Q33. Please select the reason why you chose “No” from the following options.<br>・こうした話し合いを子どもが嫌がると思うから<br>I think my child would not like these discussions.<br>・結局判断を任されるから<br>I'm left to make the decision in the end.<br>・親が意思決定すべきだから<br>Parents should make decisions.<br>・関心がなさそうだから<br>My child doesn't seem interested.<br>・説明したら、接種したくないと言いそうだから<br>I don't think my child would want to be vaccinated if I explained it to her.<br>・関係がギクシャクしそうだから<br>The relationship is likely to be strained.

	・子どもと話しにくいため<br>I am reluctant to talk to my child.<br>・その他<br>Other.
35.	子どもに子宮頸がんのワクチン接種を「する」ことで、どんなメリット（いいこと）があると、あなたは考えていますか。それぞれの項目に、最もあてはまるものをお答えください。（7件法）<br>What do you think are the advantages (good things) of “vaccinating” your child against cervical cancer? For each item, please answer the one that best applies. (7-point scale)
	将来子どもが子宮頸がんになるのを予防できる（余命が伸びる）<br>Prevents children from developing cervical cancer in the future (increased life expectancy).
	悩み事が減る（心理的負担の軽減）<br>Reduced worries (reduced psychological burden).
	将来子どもの日常生活（仕事・将来の家庭）に支障をきたさずにいられる<br>It will not interfere with my child's future daily life (work and future family).
	経済的負担の軽減に繋がる<br>It leads to a reduction in financial burden.

	周囲の接種状況に孤立せずに済む<br><br>I can be less isolated from the vaccination situation around me.
36.	子どもに子宮頸がんのワクチン接種を「する」ことで、どんなデメリット（悪いこと）があると、あなたは考えていますか。それぞれの項目に、最もあてはまるものをお答えください。（7 件法）<br><br>What do you think are the disadvantages (bad things) of “vaccinating” your child against cervical cancer? For each item, please answer the one that applies best to you. (7-point scale)
	副反応が起こるかもしれない<br><br>Adverse reactions may occur.
	子どもに後遺症が残った場合に、自責感に苛まれる<br><br>Feelings of self-blame if my child is left with sequelae.
	異常があった際に「親のせいだ」と子どもに言われるのが辛い<br><br>Difficulties with children saying 'it's the parents' fault' when there is an abnormality.
	問題発生時に子どもとの関係が悪くなる<br><br>Relationships with my child deteriorate when problems occur.
	子どもの意思を尊重できないかもしれない<br><br>May not respect my child's wishes

37.	子どもに子宮頸がんのワクチン接種を「しない」ことで、どんなメリット（いいこと）があると、あなたは考えていますか。それぞれの項目に、最も あてはまるものをお答えください。（7 件法）<br>What do you think are the advantages (good things) of "not vaccinating" children against cervical cancer? For each item, please answer the one that best applies. (7-point scale)
	副反応で子どもを苦しめるリスクを回避できる<br>Avoids the risk of my child suffering from adverse reactions.
	子どもの人生にマイナスの影響を与えないため、自責感に苛まれずに済む<br>I can avoid feelings of self-blame because I can avoid negative impact on my child's life.
	子どもとの関係を崩さずに維持できる<br>I can maintain a relationship with my child without disruption.
	子どもや配偶者との相談の手間が省ける<br>I can avoid the need to consult with my child or partner.
	意思決定の負担を負わずに済む<br>I can avoid the burden of decision-making.

38.	子どもに子宮頸がんのワクチン接種を「しない」ことで、どんなデメリット（悪いこと）があると、あなたは考えていますか。それぞれの項目に、最もあてはまるものをお答えください。（7 件法）<br>What do you think are the disadvantages (bad things) of “not vaccinating” your child against cervical cancer? For each item, please answer the one that applies best to you. (7-point scale)
	子宮喪失・妊孕性喪失の可能性や余命が短くなる可能性がある<br>Possibility of loss of uterus/fertility and shortened life expectancy.
	将来がんが発見された際に、接種しない選択をしたことを後悔する（自分を責める）<br>Regret (blame yourself) for choosing not to vaccinate if cancer is detected in the future.
	ワクチンを無料で受けるチャンスを失う／将来的な治療費がかさむ<br>Losing the chance to receive the vaccine free of charge / higher future treatment costs.
	ワクチンを受けさせなかったことにより、子どもから反感を買う<br>I am antagonized by my child for not getting the vaccine.
	接種していないことで周りから責められる<br>I am blamed by others for not vaccinating.
39.	これから 1 年以内に子宮頸がん検診を受診する予定はありますか？(2 件法)

	Do you plan to undergo cervical cancer screening within the next 12 months? (6-point scale)
40.	今までに，子宮頸がん検診を受診したことはありますか？最近受診した<br><br>時期を１つだけお答えください。<br><br>Have you ever had a cervical cancer screening before? Please answer only one question about when you were most recently examined.<br><br>・最近１年以内に受診した<br><br>Had a check-up within the last one year.<br><br>・最近２年以内に受診した<br><br>Had a check-up within the last two years.<br><br>・２年より前に受診したことがある<br><br>Had a check-up before 2 years.<br><br>・子宮頸がん検診を受診したことがない<br><br>Never.<br><br>・わからない・把握していない<br><br>Don't know/not aware of it.
41.	子宮頸がんを予防するために子どもに子宮頸がん検診を受けさせると思<br><br>いますか？（６件法）<br><br>Do you think you would have your child undergo cervical cancer screening to prevent cervical cancer? (6-point scale)

42.	子宮頸がんの検診を子どもに「受けさせる」ことで、どんなメリット（いいこと）があると、あなたは考えていますか？それぞれの項目に、最も当てはまるものをお答えください。（7 件法）<br>What do you think are the advantages (good things) of “letting” your child undergo screening for cervical cancer? For each item, please answer the one that best applies. (7-point scale)
	障害や症状への付き合い方を知ることができる<br>I can know how to deal with disorders and symptoms.
	がんの早期発見・予防に繋がる<br>It can lead to early detection and prevention of cancer.
	検診を受けることで、周りの人と繋がり相談できるようになる<br>Having a check-up enables me to connect with and discuss the issue with those around me.
	がん検診に定期的に行くことで、子どもが健康であると安心し続けることができる（心理的負担の軽減）<br>Regularly going for cancer screening can continue to reassure me that my child is healthy (reduced psychological burden).
	将来の経済的負担を減らすことができる<br>It can reduce future financial burdens.

43.	子宮頸がんの検診を子どもに「受けさせる」ことで、どんなデメリット（悪いこと）があると、あなたは考えていますか？それぞれの項目に、最も当てはまるものをお答えください。（7 件法）<br>What do you think are the disadvantages (bad things) of “letting” your child get screened for cervical cancer? For each item, please answer the one that applies most to you. (7-point scale)
	受診することで病気に関する不安に向き合わねばいけなくなる<br>I have to face the anxiety associated with the disease by going for a medical examination.
	検査によって身体に負担がかかる<br>The tests put a strain on the body.
	一度では効果がないため、継続的に受けさせることで、子どもの治療意欲を低下させる<br>Continuous testing reduces children's willingness to undergo treatment, as it is not effective once.
	もしがんが見つかったら、膨大な時間を治療のために使い、今後の人生設計に影響する<br>If cancer is found, a huge amount of time is spent on treatment, affecting future life planning.
	子どもから嫌がられる<br>My child will dislike me.

44.	子宮頸がんの検診を子どもに「受けさせない」ことで、どんなメリット（いいこと）があると、あなたは考えていますか？それぞれの項目に、最も当てはまるものをお答えください。（7 件法）<br>What do you think are the advantages (good things) of “not letting” your child be screened for cervical cancer? For each item, please answer the one that best applies. (7-point scale)
	検診・ワクチンを受けなくて済むため、子どもへの負担をかけずに済む<br>Not having to undergo check-ups/vaccinations, thus putting less strain on my child.
	不安に向き合わずに済む<br>No need to face anxiety.
	これまでと同じ生活を自由に送ることができる<br>Free to lead the same life as before.
	子どもとの関係を現状維持できる<br>I can maintain the status quo in my relationship with my child.
	心配性だと思われずに済む<br>Not be perceived as a worrier.
45.	子宮頸がんの検診を子どもに「受けさせない」ことで、どんなデメリット（悪いこと）があると、あなたは考えていますか？それぞれの項目に、最も当てはまるものをお答えください。（7 件法）

	What do you think are the disadvantages (bad things) of "not letting" your child be screened for cervical cancer? For each item, please answer the one that applies most to you. (7-point scale)
	子宮喪失・妊孕性喪失の可能性や余命が短くなる可能性がある<br>Possibility of loss of uterus and fertility and shortened life expectancy.
	将来への不安感が低減せず、不安に苛まれる<br>Unreduced sense of uncertainty and insecurity about her future.
	長期間の治療が必要になり、治療費が高額になる<br>Longer treatment required and higher treatment costs.
	子どもに検診を受けさせなかったことにより、のちのち子どもから反感を買うかもしれない<br>Failure to allow my child to undergo check-ups may later be resented by her.
	周りの流れから孤立する<br>Isolated from the flow of life around me.
46.	健康に関するリテラシー・信念についてお伺いします。それぞれの項目に、最も当てはまるものをお答えください。（6 件法）<br>We would like to ask you about your health literacy beliefs. For each item, please answer the one that applies most to you. (6-point scale)
	集団免疫が獲得されることで感染が収束すると思う<br>I believe that the acquisition of herd immunity will bring the infection to an end.

	日本において新型コロナウイルス感染症の死亡率が低いのは BCG ワクチン（BacilleCalmette-Guerin）のおかげであると思う<br>I think that the low mortality rate of COVID-19 infection in Japan is due to the BCG vaccine (BacilleCalmette-Guerin).
	とにかく３密（密閉・密集・密接）を避けることが重要だと思う<br>I think it is important to avoid “Three Cs” (closed space, crowded places, and close-contact settings).
	日本人は新型コロナウイルスに特別かかりにくいと思う<br>I think the Japanese are particularly susceptible to COVID-19.
	日本でも欧米のような感染爆発が起こっていたと思う<br>I think there has been an explosion of infections in Japan like in the West.
	子宮頸がんのワクチンにより子宮頸がんで亡くなる人を減らすことができる<br>A vaccine for cervical cancer could reduce the number of people dying from cervical cancer.
	新型コロナウイルスのワクチンが開発されれば、それを接種すれば不安はなくなると思う<br>If a vaccine for COVID-19 is developed, I think that vaccination against it would eliminate my fears.

	新型コロナウイルスのワクチンで副反応が明らかとなったとしても、それはあまり気にならないだろう<br>If adverse reactions were evident with COVID-19 vaccine, they would not be of much concern.
	子宮頸がん（HPV） ワクチンの副反応（比較的軽症度だと頭痛、筋肉痛、倦怠感など、重症度だとアナフィラキシーなど）が新型コロナウイルスのワクチンでも見られるということが明らかになったとしても、コロナウイルスのワクチンを接種すると思う<br>Even if it became clear that adverse reactions to the cervical cancer (HPV) vaccine (headache, muscle pain, and fatigue in relatively mild cases and anaphylaxis in more severe cases) were also seen with COVID-19 vaccine, I would still get the vaccine.
47.	あなたは健康に関する情報を主にどのような手段で取得していますか？以下の中から選択してください。（複数選択可能）<br>What are the main means by which you obtain information about your health? Please select from the following. (Multiple choices possible)<br>・自治体や保健所からの通知<br>Notification from local authorities or health centers.<br>・学校・職場からの通知<br>Notification from school or workplace.<br>・自治体や保健所など地域の公的機関のホームページ

	Websites of local public institutions such as municipalities and health centers.<br>・世界保健機構（WHO）や厚生労働省、学会等の公的機関のホームページ<br>Websites of public institutions such as the World Health Organization (WHO), Ministry of Health, Labour and Welfare, and academic societies.<br>・新聞やテレビ、雑誌の報道<br>Newspaper, television and magazine reports.<br>・かかりつけの医師など知り合いの医療者の助言<br>Advice from family doctors and other medical professionals.<br>・家族の意見<br>Opinions of family members.<br>・周囲の友人の意見<br>Opinions of surrounding friends.<br>・SNS やウェブサイトでの情報・評判<br>Information and reputation on SNS and websites.<br>・経験者のブログなどの体験談<br>Stories from blogs and other sources of experience.<br>・その他（）<br>Other.<br>・特になし

	None in particular.
48.	健康に関する情報はどのくらいの頻度で確認しますか？<br><br>How often do you check information about your health?<br><br>・1 日数回以上<br><br>Several times a day or more.<br><br>・ 1 日 1 回<br><br>Once a day<br><br>・週に数回<br><br>Several times a week<br><br>・週に数回未満<br><br>Less than a few times a week<br><br>・月に数回<br><br>A few times a month<br><br>・ほとんど見ない<br><br>Hardly ever<br><br>・その他<br><br>Other
49.	将来、新型コロナウイルスの予防ワクチンができた際に、あなたがそれを子どもに接種させるかどうかを判断する際に参考とする情報を以下の中から選択してください。(Multiple selections)

	When a vaccine to prevent COVID-19 becomes available, please select the information below that you would use to help you decide whether or not to give it to your child.<br>・自治体や保健所からの通知<br>Notification from local authorities or health centers.<br>・学校・職場からの通知<br>Notification from school or workplace.<br>・自治体や保健所など地域の公的機関のホームページ<br>Websites of local public institutions such as municipalities and health centers.<br>・世界保健機構（WHO）や厚生労働省、学会等の公的機関のホームページ<br>Websites of public institutions such as the World Health Organization (WHO), Ministry of Health, Labour and Welfare, and academic societies.<br>・新聞やテレビ、雑誌の報道<br>Newspaper, television and magazine reports.<br>・かかりつけの医師など知り合いの医療者の助言<br>Advice from family doctors and other medical professionals.<br>・家族の意見<br>Opinions of family members.<br>・周囲の友人の意見<br>Opinions of surrounding friends.

	・SNS やウェブサイトでの情報・評判<br><br>Information and reputation on SNS and websites.<br><br>・経験者のブログなどの体験談<br><br>Stories from blogs and other sources of experience.<br><br>・その他（）<br><br>Other.<br><br>・特になし<br><br>None in particular.
50.	子宮頸がん（HPV）のワクチンを子どもに接種させるかどうかを判断する際に参考とする情報を以下の中から選択してください。(Multiple selections)<br>Please select from the following information to help you decide whether to vaccinate your child against cervical cancer (HPV).<br><br>・自治体や保健所からの通知<br><br>Notification from local authorities or health centers.<br><br>・学校・職場からの通知<br><br>Notification from school or workplace.<br><br>・自治体や保健所など地域の公的機関のホームページ<br><br>Websites of local public institutions such as municipalities and health centers.

	・世界保健機構（WHO）や厚生労働省、学会等の公的機関のホームページ<br>Websites of public institutions such as the World Health Organization (WHO), Ministry of Health, Labour and Welfare, and academic societies.<br>・新聞やテレビ、雑誌の報道<br>Newspaper, television and magazine reports.<br>・かかりつけの医師など知り合いの医療者の助言<br>Advice from family doctors and other medical professionals.<br>・家族の意見<br>Opinions of family members.<br>・周囲の友人の意見<br>Opinions of surrounding friends.<br>・SNS やウェブサイトでの情報・評判<br>Information and reputation on SNS and websites.<br>・経験者のブログなどの体験談<br>Stories from blogs and other sources of experience.<br>・その他（）<br>Other.<br>・特になし<br>None in particular.

51.	信用している情報源を以下の中から選択してください。（複数選択可能）<br><br>Please select the sources of information you trust from the following. (Multiple selections)<br><br>・自治体や保健所からの通知<br><br>Notification from local authorities or health centers.<br><br>・学校・職場からの通知<br><br>Notification from school or workplace.<br><br>・自治体や保健所など地域の公的機関のホームページ<br><br>Websites of local public institutions such as municipalities and health centers.<br><br>・世界保健機構（WHO）や厚生労働省、学会等の公的機関のホームページ<br><br>Websites of public institutions such as the World Health Organization (WHO), Ministry of Health, Labour and Welfare, and academic societies.<br><br>・新聞やテレビ、雑誌の報道<br><br>Newspaper, television and magazine reports.<br><br>・かかりつけの医師など知り合いの医療者の助言<br><br>Advice from family doctors and other medical professionals.<br><br>・家族の意見<br><br>Opinions of family members.<br><br>・周囲の友人の意見

	Opinions of surrounding friends.<br><br>・SNS やウェブサイトでの情報・評判<br><br>Information and reputation on SNS and websites.<br><br>・経験者のブログなどの体験談<br><br>Stories from blogs and other sources of experience.<br><br>・その他（）<br><br>Other.<br><br>・特になし<br><br>None in particular.
52.	Have you ever shared or spread this information with others? (Multiple choice)<br><br>・家族や友人などに直接会って話した・電話した・メールした・メッセージアプリ（LINE など）で共有した<br><br>Shared it with family and friends in person, by phone, email or messaging app (e.g. LINE)<br><br>・SNS に投稿した<br><br>Posted on social networking sites.<br><br>・SNS で流れてきた情報を拡散した<br><br>Spread the information on SNS.<br><br>・ブログなど、SNS 以外のインターネット上に投稿した<br><br>Posted on the internet other than SNS, e.g. blogs.<br><br>・その他の方法で拡散した

	Spread the information in other ways.<br>・共有・拡散していない<br>Didn’t share or spread the information.<br>・わからない<br>Don't know.
53.	The Hong Psychological Reactance Scale (14-item 5-point scale) [1]
54.	The Japanese version of the Perceived Vulnerability to Disease Scale (15-item 5-point scale) [2]
55.	The neurogenic tendencies of the Big Five (5-item 5-point scale). [3]

For men

1.	あなたの性別と年齢を教えてください。<br>What is your gender and age?
2.	あなたの家族構成についてお伺いします。<br>What is your family structure?
3.	あなたが今まで接種したことのあるワクチンを教えてください。<br>Please tell us which vaccines you have been vaccinated against.
	(インフルエンザ、子宮頸がん（HPV)、三種(百日咳・ジフテリア・破傷風)混合(DPT)、日本脳炎、その他のワクチン）<br>(Influenza, cervical cancer (HPV), triad combined (diphtheria / pertussis / tetanus) (DPT), Japanese encephalitis, and other vaccines)

4.	あなたは以前がんなどの病気に罹患した経験がありますか？ある場合、その病名を教えてください。<br>Have you ever had cancer or any other disease before? If yes, what is the name of the disease?
5.	一生涯のうち一般的な日本人が以下の病気にかかる可能性はどれくらいだと思いますか？それぞれの項目に、最も当てはまるものをお答えください。（7 件法）<br>How likely do you think it is that the average Japanese person will suffer from the following diseases during their lifetime? For each item, please answer the most applicable. (7-point scale)
	(胃がん、肺がん、大腸がん、新型コロナウイルス感染症、インフルエンザ)<br>(Stomach cancer, lung cancer, colorectal cancer, new coronavirus infection, influenza)
6.	一生涯のうち自分自身が以下の病気にかかる可能性はどれくらいだと思いますか？それぞれの項目に、最も当てはまるものをお答えください。（7 件法）<br>How likely do you think you are to get any of the following diseases yourself in your lifetime? For each item, please answer the most applicable. (7-point scale)

	(胃がん、肺がん、大腸がん、新型コロナウイルス感染症、インフルエンザ)<br>(Stomach cancer, lung cancer, colorectal cancer, new coronavirus infection, influenza)
7.	一生涯のうちあなたの子どもが以下の病気にかかる可能性はどれくらいだと思いますか？それぞれの項目に、最も当てはまるものをお答えください。（7 件法）<br>How likely do you think your child is to get any of the following diseases during his or her lifetime? For each item, please answer the most applicable. (7-point scale)
	(胃がん、肺がん、大腸がん、新型コロナウイルス感染症、インフルエンザ)<br>(Stomach cancer, lung cancer, colorectal cancer, new coronavirus infection, influenza)
8.	将来あなたが以下の病気にかかることによる経済的負担や仕事などへの影響はどれくらいだと思われますか？それぞれの項目に、最も当てはまるものをお答えください。（7 件法）<br>How much do you think the financial burden and impact on your work would be if you were to suffer from any of the following diseases in the future? For each item, please answer the most applicable.(7-point scale)

	(胃がん、肺がん、大腸がん、新型コロナウイルス感染症、インフルエンザ)<br>(Stomach cancer, lung cancer, colorectal cancer, new coronavirus infection, influenza)
9.	将来あなたの子どもが以下の病気にかかることによる経済的負担や仕事などへの影響はどれくらいだと思われますか？それぞれの項目に、最も当てはまるものをお答えください。（7 件法）<br>How much do you think the financial burden and impact on your work would be if your child were to suffer from any of the following diseases in the future? For each item, please answer the most applicable. (7-point scale)
	(胃がん、肺がん、大腸がん、新型コロナウイルス感染症、インフルエンザ)<br>(Stomach cancer, lung cancer, colorectal cancer, new coronavirus infection, influenza)
10.	あなたは新型コロナウイルスについて、以下の情報を知っていますか。それぞれの項目に、最も当てはまるものをお答えください。（6 件法）<br>Do you know about the following information on COVID-19? For each item, please answer the one that best applies. (6-point scale)
	・コロナウイルスは遺伝情報として RNA ウイルスの一種（一本鎖 RNA ウイルス）である

	Coronaviruses are a type of RNA virus (single-stranded RNA virus) with genetic information
	・一般的には飛沫感染、接触感染で感染する<br><br>Generally transmitted by droplet or contact infection.
	・咳や咽頭通などの症状が明らかになる前から、感染が広がるおそれがあると言われている<br><br>It is said that the infection may spread even before symptoms such as coughing and pharyngeal discharge become apparent.
	・マスク着用、手洗いやアルコールによる消毒などが感染の予防に重要である<br><br>The use of masks, hand washing and disinfection with alcohol are important to prevent infection.
	・感染の有無を調べるには PCR 検査などがある<br><br>PCR tests and other tests are available to determine the presence of infection.
11.	新型コロナウイルス予防ワクチンの開発についての報道をご存知ですか？（2 件法）<br><br>Are you aware of any media reports about the development of a vaccine to prevent new coronaviruses? (2-point scale)
12.	新型コロナウイルス予防ワクチンの開発は成功し、実施されるようになると思いますか？（2 件法）

	Do you think the development of a vaccine to prevent COVID-19 will be successful and become implemented? (2-point scale)
13.	新型コロナウイルスの予防など新型コロナウイルス対策のワクチンは信頼できると思いますか？（6 件法）<br>Do you think that vaccine against COVID-19 is reliable? (6-point scale)
14.	以下のような条件下で、あなたは新型コロナウイルスを予防するためのワクチンを接種しようと思いますか？（6 件法）<br>Under the following conditions, would you be willing to be vaccinated to prevent COVID-19? (6-point scale)
	副作用などリスクがまったくない場合<br>If there are no side effects or other risks at all.
	痛みやアレルギー反応などインフルエンザワクチンなどすでに普及しているワクチンと同程度の副作用のリスクがある場合<br>If there is a risk of side-effects comparable to those of vaccines already in widespread use, such as influenza vaccines, including pain and allergic reactions.
	まだ明らかになっていない副作用などのリスクがある場合<br>If there is a risk of side-effects or other risks that have not yet been identified.
15.	以下のような条件下で、あなたは新型コロナウイルスを予防するためのワクチンを「子ども」に接種させると思いますか？（6 件法）

	Under the following conditions, would you allow your **child** to be vaccinated against COVID-19? (6-point scale)
	副作用などリスクがまったくない場合<br>If there are no side effects or other risks at all.
	痛みやアレルギー反応などインフルエンザワクチンなどすでに普及しているワクチンと同程度の副作用のリスクがある場合<br>If there is a risk of side-effects comparable to those of vaccines already in widespread use, such as influenza vaccines, including pain and allergic reactions.
	まだ明らかになっていない副作用などのリスクがある場合<br>If there is a risk of side-effects or other risks that have not yet been identified.
16.	有効な治療法が開発されたとしても、ワクチンなどを使って新型コロナウイルスを予防することは大事だと思いますか？（6 件法）<br>Even if an effective treatment is developed, do you think it is important to prevent COVID-19 through vaccines or other means? (6-point scale)
17.	配偶者（パートナー）がいる方に質問します。子どもへの新型コロナウイルスのワクチン接種について配偶者と検討したことがある、あるいは、する予定はありますか？（2 件法）<br>Question for those with a spouse (partner). Have you considered or do you plan to consider vaccinating your children against COVID-19 with your spouse? (2-point scale)

18.	問 17 で「いいえ」を選択した方に伺います。「いいえ」を選択した理由を以下から選択してください。<br>If you selected “No” in Q17. Please select the reason why you chose “No” from the following options.<br>・そこまで重要だと考えていないから<br>I don't think it is that important.<br>・子どもの選択であるから<br>It is my child's choice.<br>・相談してもしょうがないから<br>There's no point in discussing it.<br>・情報共有のみで良いと思うから<br>I think it's good to just share information.<br>・相談しても聞いてもらえないだろうから<br>My partner wouldn't talk to me about it and wouldn't listen to me.<br>・その他<br>Other.
19.	新型コロナウイルスのワクチン接種について子どもと話し合ったことがありますか？（2 件法）<br>Have you ever discussed vaccination against the new coronavirus with your child? (2-point scale)

20.	問 19 で「いいえ」を選択した方に伺います。「いいえ」を選択した理由を以下から選択してください。<br>If you selected “No” in Q19. Please select the reason why you chose “No” from the following options.<br>・こうした話し合いを子どもが嫌がると思うから<br>I think my child would not like these discussions.<br>・結局判断を任されるから<br>I'm left to make the decision in the end.<br>・親が意思決定すべきだから<br>Parents should make decisions.<br>・関心がなさそうだから<br>My child doesn't seem interested.<br>・説明したら、接種したくないと言いそうだから<br>I don't think my child would want to be vaccinated if I explained it to her.<br>・関係がギクシャクしそうだから<br>The relationship is likely to be strained.<br>・子どもと話しにくいため<br>I am reluctant to talk to my children.<br>・その他<br>Other.

21.	子どもに新型コロナウイルスのワクチン接種を「する」ことで、どんなメリット（いいこと）があると、あなたは考えていますか。それぞれの項目に、最も あてはまるものをお答えください。（7 件法）<br>What do you think are the benefits (good things) of “vaccinating” your child against COVID-19? For each item, please answer the one that best applies. (7-point scale)
	感染を防ぐことができる<br>Able to prevent infection.
	入院や隔離を回避できる<br>Avoid hospitalisation or isolation.
	家族や職場に迷惑をかけなくて済む<br>No more inconvenience to family or work place.
	周りの人と同じ行動を取ることで安心できる<br>I feel safe in behaving in the same way as others around me.
	周囲の人に不快な思いをさせない<br>Not causing discomfort to those around me.
22.	子どもに新型コロナウイルスのワクチン接種を「する」ことで、どんなデメリット（悪いこと）があると、あなたは考えていますか。それぞれの項目に、最もあてはまるものをお答えください。（7 件法）

	What do you think are the disadvantages (bad things) of “vaccinating” your child against COVID-19? For each item, please answer the one that applies best to you. (7-point scale)
	副反応が起こるかもしれない<br><br>Adverse reactions might occur.
	子どもに後遺症が残った場合に、自責感に苛まれる<br><br>Feeling self-condemned if my child is permanently injured.
	異常があった際に「親のせいだ」と子どもに言われるのが辛い<br><br>It is hard for children to be told that it is their parents' fault when there is an abnormality.
	問題発生時に子どもとの関係が悪くなる<br><br>Poor relationship with my child when problems occur.
	ワクチン接種しても効果がない可能性がある<br><br>Vaccination may not be effective.
23.	子どもに新型コロナウイルスのワクチン接種を「しない」ことで、どんなメリット（いいこと）があると、あなたは考えていますか。それぞれの項目に、最もあてはまるものをお答えください。（7 件法）<br><br>What do you think are the advantages (good things) of “not vaccinating” your child against the new coronavirus? For each item, please answer the one that best applies. (7-point scale)
	副反応で子どもを苦しめるリスクを回避できる<br><br>Avoid the risk of my child suffering from adverse reactions.

	意思決定の負担を負わずに済む<br><br>Not having to bear the burden of decision-making.
	子どもの人生に責任を持たずに済む<br><br>I don't have to take responsibility for my child's life.
	これまで通りでいられるため、ストレスが少ない<br><br>Less stressful as it can be business as usual.
	ワクチンによる問題が発生しないため、子どもとの関係を崩さずに維持できる<br><br>No problems or changes caused by the vaccine, so the relationship with my child can be maintained without disruption.
24.	子どもに新型コロナウイルスのワクチン接種を「しない」ことで、どんなデメリット（悪いこと）があると、あなたは考えていますか。それぞれの項目に、最もあてはまるものをお答えください。（7 件法）<br><br>What do you think are the disadvantages (bad things) of “not vaccinating” your child against the new coronavirus? For each item, please answer the one that applies best to you. (7-point scale)
	感染を防ぐことができず、感染リスクが高いままになる<br><br>Failure to prevent infection, leaving the patient at high risk of infection.
	入院や隔離となるリスクを抱えたままとなる<br><br>Remain at risk of hospitalization or quarantine.
	職場や家族に迷惑をかけるかもしれない

	May cause problems at work or with family members.
	周りの人から白い目で見られる<br><br>People around you look at you in a white way.
	周囲の人に不快な思いをさせる<br><br>Makes people around you feel uncomfortable.
25.	あなたは子宮頸がんについて以下の情報を知っていますか？それぞれの<br><br>項目に、最も当てはまるものをお答えください。（6 件法）<br><br>Are you aware of the following information about cervical cancer? For each item, please answer the one that best applies. (6-point scale)
	遺伝情報として DNA ウイルスの一種（環状二本鎖 DNA ウイルス）で<br><br>ある<br><br>It is a type of DNA virus (cyclic double-stranded DNA virus) with genetic information.
	主に性交渉で感染する<br><br>Mainly sexually transmitted.
	感染しても症状は現れず、症状のないまま感染が広がる<br><br>Infection does not cause symptoms and spreads without symptoms.
	コンドームは感染予防に有効ではあるが、感染を完全に防ぐことはでき<br><br>ない<br><br>Condoms are effective in preventing infection, but do not prevent infection altogether.

	感染の有無を調べるには、ハイブリッドキャプチャー法や PCR 法などがある<br>Hybrid capture and PCR methods are used to test for the presence of infection.
26.	子宮頸がん予防ワクチンの副反応についての報道をご存知ですか？（2 件法）<br>Are you aware of any media reports about adverse reactions to the cervical cancer prevention vaccine? (2-point scale)
27.	子宮頸がん予防ワクチンの安全性に問題があると思いますか？（6 件法）<br>Do you think there are safety issues with the cervical cancer prevention vaccine? (6-point scale)
28.	子宮頸がんを予防するために子どもにワクチン接種をさせると思いますか？(選択)<br>Would you have your child vaccinated to prevent cervical cancer? (Select one)<br>・勧奨が再開されなくても、接種させる<br>Even if the recommendation is not reintroduced, my child should be vaccinated.<br>・勧奨が開始されたら、接種させる<br>I will have my child vaccinated when the recommendation is launched.

	・自分の周りや知り合いが接種してから接種させる<br>I would make my children vaccinate after I and others I know have been vaccinated.<br>・同世代の多くの子が接種していることが分かってから接種させる<br>I would have my child vaccinated only after I knew that many children of my generation had been vaccinated.<br>・接種させない<br>Not vaccinate.<br>・決められない<br>I can't make up my mind.<br>・その他<br>Other
29.	以下のような条件下で、あなたは子宮頸がんを予防するためのワクチンを「子ども」に接種させると思いますか？それぞれの項目に、最も当てはまるものをお答えください。（6 件法）<br>Under the following conditions, would you allow “your child” to be vaccinated to prevent cervical cancer? For each item, please answer the one that best applies. (6-point scale)
	副作用などリスクがまったくない場合<br>If there are no side effects or other risks at all.

	痛みやアレルギー反応などインフルエンザワクチンなどすでに普及しているワクチンと同程度の副作用のリスクがある場合<br>If there is a risk of side effects comparable to those of vaccines already in widespread use, such as influenza vaccines, including pain and allergic reactions.
	重篤な副反応などのリスク（0.01%程度）がある場合<br>Risk of serious adverse reactions and other reactions (around 0.01%).
30.	ワクチンを使って、子宮頸がんを予防することは大事だと思いますか？（6 件法）<br>Do you think it is important to use vaccines to prevent cervical cancer? (6-point scale)
31.	配偶者（パートナー）がいる方に質問します。子どもへの子宮頸がん（HPV）ワクチン接種について配偶者と検討したことがある、あるいは、する予定はありますか？ （2 件法）<br>Question for those with a spouse (partner). Have you ever considered or do you plan to consider vaccinating your children against cervical cancer (HPV) with your spouse? (2-point scale)
32.	問 31 で「いいえ」を選択した方に伺います。「いいえ」を選択した理由を以下から選択してください。<br>if you selected “No” in Q31. Please select the reason why you chose “No” from the following options.

	・そこまで重要だと考えていないから<br><br>I don't think it is that important.<br><br>・子どもの選択であるから<br><br>It is my child's choice.<br><br>・相談してもしょうがないから<br><br>There's no point in discussing it<br><br>・情報共有のみで良いと思うから<br><br>I think it's good to just share information.<br><br>・相談しても聞いてもらえないだろうから<br><br>My partner wouldn't talk to me about it and wouldn't listen to me.<br><br>・その他<br><br>Other
33.	子宮頸がん（HPV）ワクチン接種について子どもと話し合ったことがありますか？（2 件法）<br><br>Have you ever discussed cervical cancer (HPV) vaccination with your child? (2-point scale)
34.	問 33 で「いいえ」を選択した方に伺います。「いいえ」を選択した理由を以下から選択してください。<br><br>if you selected “No” in Q33. Please select the reason why you chose “No” from the following options.<br><br>・こうした話し合いを子どもが嫌がると思うから

	I think my child would not like these discussions.<br>・結局判断を任されるから<br>I'm left to make the decision in the end.<br>・親が意思決定すべきだから<br>Parents should make decisions.<br>・関心がなさそうだから<br>My child doesn't seem interested.<br>・説明したら、接種したくないと言いそうだから<br>I don't think my child would want to be vaccinated if I explained it to her.<br>・関係がギクシャクしそうだから<br>The relationship is likely to be strained.<br>・子どもと話しにくいため<br>I am reluctant to talk to my child.<br>・その他<br>Other.
35.	子どもに子宮頸がんのワクチン接種を「する」ことで、どんなメリット（いいこと）があると、あなたは考えていますか。それぞれの項目に、最もあてはまるものをお答えください。（7 件法）<br>What do you think are the advantages (good things) of “vaccinating” your child against cervical cancer? For each item, please answer the one that best applies. (7-point scale)

	将来子どもが子宮頸がんになるのを予防できる（余命が伸びる）<br><br>Prevents children from developing cervical cancer in the future (increased life expectancy).
	悩み事が減る（心理的負担の軽減）<br><br>Reduced worries (reduced psychological burden).
	将来子どもの日常生活（仕事・将来の家庭）に支障をきたさずにいられる<br><br>It will not interfere with my child's future daily life (work and future family).
	経済的負担の軽減に繋がる<br><br>It leads to a reduction in financial burden.
	周囲の接種状況に孤立せずに済む<br><br>I can be less isolated from the vaccination situation around me.
36.	子どもに子宮頸がんのワクチン接種を「する」ことで、どんなデメリット（悪いこと）があると、あなたは考えていますか。それぞれの項目に、最もあてはまるものをお答えください。（7件法）<br><br>What do you think are the disadvantages (bad things) of “vaccinating” your child against cervical cancer? For each item, please answer the one that applies best to you. (7-point scale)
	副反応が起こるかもしれない<br><br>Adverse reactions may occur.

	子どもに後遺症が残った場合に、自責感に苛まれる<br><br>Feelings of self-blame if my child is left with sequelae.
	異常があった際に「親のせいだ」と子どもに言われるのが辛い<br><br>Difficulties with children saying 'it's the parents' fault' when there is an abnormality.
	問題発生時に子どもとの関係が悪くなる<br><br>Relationships with my child deteriorate when problems occur.
	子どもの意思を尊重できないかもしれない<br><br>May not respect my child's wishes
37.	子どもに子宮頸がんのワクチン接種を「しない」ことで、どんなメリット（いいこと）があると、あなたは考えていますか。それぞれの項目に、最もあてはまるものをお答えください。（7 件法）<br><br>What do you think are the advantages (good things) of “not vaccinating” children against cervical cancer? For each item, please answer the one that best applies. (7-point scale)
	副反応で子どもを苦しめるリスクを回避できる<br><br>Avoids the risk of my child suffering from adverse reactions.
	子どもの人生にマイナスの影響を与えないため、自責感に苛まれずに済む<br><br>I can avoid feelings of self-blame because I can avoid negative impact on my child's life.

	子どもとの関係を崩さずに維持できる<br><br>I can maintain a relationship with my child without disruption.
	子どもや配偶者との相談の手間が省ける<br><br>I can avoid the need to consult with my child or partner.
	意思決定の負担を負わずに済む<br><br>I can avoid the burden of decision-making.
38.	子どもに子宮頸がんのワクチン接種を「しない」ことで、どんなデメリット（悪いこと）があると、あなたは考えていますか。それぞれの項目に、最もあてはまるものをお答えください。（7 件法）<br><br>What do you think are the disadvantages (bad things) of “not vaccinating” your child against cervical cancer? For each item, please answer the one that applies best to you. (7-point scale)
	子宮喪失・妊孕性喪失の可能性や余命が短くなる可能性がある<br><br>Possibility of loss of uterus/fertility and shortened life expectancy.
	将来がんが発見された際に、接種しない選択をしたことを後悔する（自分を責める）<br><br>Regret (blame yourself) for choosing not to vaccinate if cancer is detected in the future.
	ワクチンを無料で受けるチャンスを失う／将来的な治療費がかさむ<br><br>Losing the chance to receive the vaccine free of charge / higher future treatment costs.

	ワクチンを受けさせなかったことにより、子どもから反感を買う<br><br>I am antagonized by my child for not getting the vaccine.
	接種していないことで周りから責められる<br><br>I am blamed by others for not vaccinating.
39.	これから1年以内に自身で何かしらのがん検診を受診する予定はありますか？(2件法)<br><br>Do you plan to undergo any kind of cancer screening yourself within the next 12 months? (6-point scale)
40.	今までに，何らかのがん検診を受診したことはありますか？最近受診した時期を1つだけお答えください。<br><br>Have you ever had any kind of cancer screening before? Please answer only one question about when you were most recently examined.<br><br>・最近1年以内に受診した<br><br>Had a check-up within the last one year.<br><br>・最近2年以内に受診した<br><br>Had a check-up within the last two years.<br><br>・２年より前に受診したことがある<br><br>Had a check-up before 2 years.<br><br>・子宮頸がん検診を受診したことがない<br><br>Never.<br><br>・わからない・把握していない

	Don't know/not aware of it.
41.	子宮頸がんを予防するために子どもに子宮頸がん検診を受けさせると思いますか？（6 件法）<br>Do you think you would have your child undergo cervical cancer screening to prevent cervical cancer? (6-point scale)
42.	子宮頸がんの検診を子どもに「受けさせる」ことで、どんなメリット（いいこと）があると、あなたは考えていますか？それぞれの項目に、最も当てはまるものをお答えください。（7 件法）<br>What do you think are the advantages (good things) of “letting” your child undergo screening for cervical cancer? For each item, please answer the one that best applies. (7-point scale)
	障害や症状への付き合い方を知ることができる<br>I can know how to deal with disorders and symptoms.
	がんの早期発見・予防に繋がる<br>It can lead to early detection and prevention of cancer.
	検診を受けることで、周りの人と繋がり相談できるようになる<br>Having a check-up enables me to connect with and discuss the issue with those around me.
	がん検診に定期的に行くことで、子どもが健康であると安心し続けることができる（心理的負担の軽減）<br>Regularly going for cancer screening can continue to reassure me that my child is healthy (reduced psychological burden).

	将来の経済的負担を減らすことができる<br>It can reduce future financial burdens.
43.	子宮頸がんの検診を子どもに「受けさせる」ことで、どんなデメリット（悪いこと）があると、あなたは考えていますか？それぞれの項目に、最も当てはまるものをお答えください。（7件法）<br>What do you think are the disadvantages (bad things) of “letting” your child get screened for cervical cancer? For each item, please answer the one that applies most to you. (7-point scale)
	受診することで病気に関する不安に向き合わねばいけなくなる<br>I have to face the anxiety associated with the disease by going for a medical examination.
	検査によって身体に負担がかかる<br>The tests put a strain on the body.
	一度では効果がないため、継続的に受けさせることで、子どもの治療意欲を低下させる<br>Continuous testing reduces children's willingness to undergo treatment, as it is not effective once.
	もしがんが見つかったら、膨大な時間を治療のために使い、今後の人生設計に影響する<br>If cancer is found, a huge amount of time is spent on treatment, affecting future life planning.

	子どもから嫌がられる<br><br>My child will dislike me.
44.	子宮頸がんの検診を子どもに「受けさせない」ことで、どんなメリット（いいこと）があると、あなたは考えていますか？それぞれの項目に、最も当てはまるものをお答えください。（7 件法）<br><br>What do you think are the advantages (good things) of “not letting” your child be screened for cervical cancer? For each item, please answer the one that best applies. (7-point scale)
	検診・ワクチンを受けなくて済むため、子どもへの負担をかけずに済む<br><br>Not having to undergo check-ups/vaccinations, thus putting less strain on my child.
	不安に向き合わずに済む<br><br>No need to face anxiety.
	これまでと同じ生活を自由に送ることができる<br><br>Free to lead the same life as before.
	子どもとの関係を現状維持できる<br><br>I can maintain the status quo in my relationship with my child.
	心配性だと思われずに済む<br><br>Not be perceived as a worrier.

45.	子宮頸がんの検診を子どもに「受けさせない」ことで、どんなデメリット（悪いこと）があると、あなたは考えていますか？それぞれの項目に、最も当てはまるものをお答えください。（7件法）<br>What do you think are the disadvantages (bad things) of “not letting” your child be screened for cervical cancer? For each item, please answer the one that applies most to you. (7-point scale)
	子宮喪失・妊孕性喪失の可能性や余命が短くなる可能性がある<br>Possibility of loss of uterus and fertility and shortened life expectancy.
	将来への不安感が低減せず、不安に苛まれる<br>Unreduced sense of uncertainty and insecurity about her future.
	長期間の治療が必要になり、治療費が高額になる<br>Longer treatment required and higher treatment costs.
	子どもに検診を受けさせなかったことにより、のちのち子どもから反感を買うかもしれない<br>Failure to allow my child to undergo check-ups may later be resented by her.
	周りの流れから孤立する<br>Isolated from the flow of life around me.
46.	健康に関するリテラシー・信念についてお伺いします。それぞれの項目に、最も当てはまるものをお答えください。（6件法）

	We would like to ask you about your health literacy beliefs. For each item, please answer the one that applies most to you. (6-point scale)
	集団免疫が獲得されることで感染が収束すると思う<br>I believe that the acquisition of herd immunity will bring the infection to an end.
	日本において新型コロナウイルス感染症の死亡率が低いのは BCG ワクチン（BacilleCalmette-Guerin）のおかげであると思う<br>I think that the low mortality rate of COVID-19 infection in Japan is due to the BCG vaccine (BacilleCalmette-Guerin).
	とにかく３密（密閉・密集・密接）を避けることが重要だと思う<br>I think it is important to avoid “Three Cs” (closed space, crowded places, and close-contact settings).
	日本人は新型コロナウイルスに特別かかりにくいと思う<br>I think the Japanese are particularly susceptible to COVID-19.
	日本でも欧米のような感染爆発が起こっていたと思う<br>I think there has been an explosion of infections in Japan like in the West.
	子宮頸がんのワクチンにより子宮頸がんで亡くなる人を減らすことができる<br>A vaccine for cervical cancer could reduce the number of people dying from cervical cancer.

	新型コロナウイルスのワクチンが開発されれば、それを接種すれば不安はなくなると思う<br>If a vaccine for COVID-19 is developed, I think that vaccination against it would eliminate my fears.
	新型コロナウイルスのワクチンで副反応が明らかとなったとしても、それはあまり気にならないだろう<br>If adverse reactions were evident with COVID-19 vaccine, they would not be of much concern.
	子宮頸がん（HPV）　ワクチンの副反応（比較的軽症度だと頭痛、筋肉痛、倦怠感など、重症度だとアナフィラキシーなど）が新型コロナウイルスのワクチンでも見られるということが明らかになったとしても、コロナウイルスのワクチンを接種すると思う<br>Even if it became clear that adverse reactions to the cervical cancer (HPV) vaccine (headache, muscle pain, and fatigue in relatively mild cases and anaphylaxis in more severe cases) were also seen with COVID-19 vaccine, I would still get the vaccine.
47.	あなたは健康に関する情報を主にどのような手段で取得していますか？以下の中から選択してください。（複数選択可能）<br>What are the main means by which you obtain information about your health? Please select from the following. (Multiple choices possible)<br>・自治体や保健所からの通知

	Notification from local authorities or health centers.<br>・学校・職場からの通知<br>Notification from school or workplace.<br>・自治体や保健所など地域の公的機関のホームページ<br>Websites of local public institutions such as municipalities and health centers.<br>・世界保健機構（WHO）や厚生労働省、学会等の公的機関のホームページ<br>Websites of public institutions such as the World Health Organization (WHO), Ministry of Health, Labour and Welfare, and academic societies.<br>・新聞やテレビ、雑誌の報道<br>Newspaper, television and magazine reports.<br>・かかりつけの医師など知り合いの医療者の助言<br>Advice from family doctors and other medical professionals.<br>・家族の意見<br>Opinions of family members.<br>・周囲の友人の意見<br>Opinions of surrounding friends.<br>・SNS やウェブサイトでの情報・評判<br>Information and reputation on SNS and websites.<br>・経験者のブログなどの体験談

	Stories from blogs and other sources of experience.<br><br>・その他（）<br><br>Other.<br><br>・特になし<br><br>None in particular.
48.	健康に関する情報はどのくらいの頻度で確認しますか？<br><br>How often do you check information about your health?<br><br>・1 日数回以上<br><br>Several times a day or more.<br><br>・ 1 日 1 回<br><br>Once a day<br><br>・週に数回<br><br>Several times a week<br><br>・週に数回未満<br><br>Less than a few times a week<br><br>・月に数回<br><br>A few times a month<br><br>・ほとんど見ない<br><br>Hardly ever<br><br>・その他<br><br>Other

49.	将来、新型コロナウイルスの予防ワクチンができた際に、あなたがそれを子どもに接種させるかどうかを判断する際に参考とする情報を以下の中から選択してください。(Multiple selections)<br>When a vaccine to prevent COVID-19 becomes available, please select the information below that you would use to help you decide whether or not to give it to your child.<br>・自治体や保健所からの通知<br>Notification from local authorities or health centers.<br>・学校・職場からの通知<br>Notification from school or workplace.<br>・自治体や保健所など地域の公的機関のホームページ<br>Websites of local public institutions such as municipalities and health centers.<br>・世界保健機構（WHO）や厚生労働省、学会等の公的機関のホームページ<br>Websites of public institutions such as the World Health Organization (WHO), Ministry of Health, Labour and Welfare, and academic societies.<br>・新聞やテレビ、雑誌の報道<br>Newspaper, television and magazine reports.<br>・かかりつけの医師など知り合いの医療者の助言<br>Advice from family doctors and other medical professionals.

	・家族の意見<br><br>Opinions of family members.<br><br>・周囲の友人の意見<br><br>Opinions of surrounding friends.<br><br>・SNS やウェブサイトでの情報・評判<br><br>Information and reputation on SNS and websites.<br><br>・経験者のブログなどの体験談<br><br>Stories from blogs and other sources of experience.<br><br>・その他（）<br><br>Other.<br><br>・特になし<br><br>None in particular.
50.	子宮頸がん（HPV）のワクチンを子どもに接種させるかどうかを判断する際に参考とする情報を以下の中から選択してください。(Multiple selections)<br>Please select from the following information to help you decide whether to vaccinate your child against cervical cancer (HPV).<br><br>・自治体や保健所からの通知<br><br>Notification from local authorities or health centers.<br><br>・学校・職場からの通知<br><br>Notification from school or workplace.

	・自治体や保健所など地域の公的機関のホームページ<br>Websites of local public institutions such as municipalities and health centers.<br>・世界保健機構（WHO）や厚生労働省、学会等の公的機関のホームページ<br>Websites of public institutions such as the World Health Organization (WHO), Ministry of Health, Labour and Welfare, and academic societies.<br>・新聞やテレビ、雑誌の報道<br>Newspaper, television and magazine reports.<br>・かかりつけの医師など知り合いの医療者の助言<br>Advice from family doctors and other medical professionals.<br>・家族の意見<br>Opinions of family members.<br>・周囲の友人の意見<br>Opinions of surrounding friends.<br>・SNS やウェブサイトでの情報・評判<br>Information and reputation on SNS and websites.<br>・経験者のブログなどの体験談<br>Stories from blogs and other sources of experience.<br>・その他（）<br>Other.

	・特になし<br><br>None in particular.
51.	信用している情報源を以下の中から選択してください。（複数選択可能）<br><br>Please select the sources of information you trust from the following. (Multiple selections)<br><br>・自治体や保健所からの通知<br><br>Notification from local authorities or health centers.<br><br>・学校・職場からの通知<br><br>Notification from school or workplace.<br><br>・自治体や保健所など地域の公的機関のホームページ<br><br>Websites of local public institutions such as municipalities and health centers.<br><br>・世界保健機構（WHO）や厚生労働省、学会等の公的機関のホームページ<br><br>Websites of public institutions such as the World Health Organization (WHO), Ministry of Health, Labour and Welfare, and academic societies.<br><br>・新聞やテレビ、雑誌の報道<br><br>Newspaper, television and magazine reports.<br><br>・かかりつけの医師など知り合いの医療者の助言<br><br>Advice from family doctors and other medical professionals.<br><br>・家族の意見

	Opinions of family members.<br><br>・周囲の友人の意見<br><br>Opinions of surrounding friends.<br><br>・SNS やウェブサイトでの情報・評判<br><br>Information and reputation on SNS and websites.<br><br>・経験者のブログなどの体験談<br><br>Stories from blogs and other sources of experience.<br><br>・その他（）<br><br>Other.<br><br>・特になし<br><br>None in particular.
52.	Have you ever shared or spread this information with others? (Multiple choice)<br><br>・家族や友人などに直接会って話した・電話した・メールした・メッセージアプリ（LINE など）で共有した<br><br>Shared it with family and friends in person, by phone, email or messaging app (e.g. LINE)<br><br>・SNS に投稿した<br><br>Posted on social networking sites.<br><br>・SNS で流れてきた情報を拡散した<br><br>Spread the information on SNS.<br><br>・ブログなど、SNS 以外のインターネット上に投稿した

	Posted on the internet other than SNS, e.g. blogs.<br>・その他の方法で拡散した<br>Spread the information in other ways.<br>・共有・拡散していない<br>Didn’t share or spread the information.<br>・わからない<br>Don't know.
53.	The Hong Psychological Reactance Scale (14-item 5-point scale) [1]
54.	The Japanese version of the Perceived Vulnerability to Disease Scale (15-item 5-point scale) [2]
55.	The neurogenic tendencies of the Big Five (5-item 5-point scale). [3]

References

1. Hong , S. M.; Page, S. A Psychological Reactance Scale: Development, Factor Structure and Reliability. Psychological Reports **1989**, 64(3), 1323-1326. [Cross-Ref]
2. Fukukawa, Y.; Oda, R.; Usami, H.; Kawahito, J. Development of a Japanese version of the Perceived Vulnerability to Disease Scale. The Japanese Journal of Psychology **2014**, 85(2), 188-195. [Cross-Ref]
3. Namikawa, T.; Tani, I.; Wakita, T.; Kumagai, R.; Nakane, A.; Noguchi, H. Development of a short form of the Japanese Big Five Scale, and a test of its reliability and validity. The Japanese Journal of Psychology 2021, 82(2), 91-99. [Cross-Ref]
